# Psychological Well-Being and the Human Conserved Transcriptional Response to Adversity

**DOI:** 10.1371/journal.pone.0121839

**Published:** 2015-03-26

**Authors:** Barbara L. Fredrickson, Karen M. Grewen, Sara B. Algoe, Ann M. Firestine, Jesusa M. G. Arevalo, Jeffrey Ma, Steve W. Cole

**Affiliations:** 1 Department of Psychology, University of North Carolina, Chapel Hill, North Carolina, United States of America; 2 Department of Psychiatry, University of North Carolina School of Medicine, Chapel Hill, North Carolina, United States of America; 3 Department of Medicine, UCLA School of Medicine, Los Angeles, California, United States of America; 4 Jonsson Comprehensive Cancer Center, UCLA School of Medicine, Los Angeles, California, United States of America; 5 Norman Cousins Center, UCLA School of Medicine, Los Angeles, California, United States of America; 6 UCLA AIDS Institute, Los Angeles, California, United States of America; 7 UCLA Molecular Biology Institute, Los Angeles, California, United States of America; University of Illinois-Urbana Champaign, UNITED STATES

## Abstract

Research in human social genomics has identified a conserved transcriptional response to adversity (CTRA) characterized by up-regulated expression of pro-inflammatory genes and down-regulated expression of Type I interferon- and antibody-related genes. This report seeks to identify the specific aspects of positive psychological well-being that oppose such effects and predict reduced CTRA gene expression. In a new confirmation study of 122 healthy adults that replicated the approach of a previously reported discovery study, mixed effect linear model analyses identified a significant inverse association between expression of CTRA indicator genes and a summary measure of eudaimonic well-being from the Mental Health Continuum – Short Form. Analyses of a 2- representation of eudaimonia converged in finding correlated psychological and social subdomains of eudaimonic well-being to be the primary carriers of CTRA associations. Hedonic well-being showed no consistent CTRA association independent of eudaimonic well-being, and summary measures integrating hedonic and eudaimonic well-being showed less stable CTRA associations than did focal measures of eudaimonia (psychological and social well-being). Similar results emerged from analyses of pooled discovery and confirmation samples (n = 198). Similar results also emerged from analyses of a second new generalization study of 107 healthy adults that included the more detailed Ryff Scales of Psychological Well-being and found this more robust measure of eudaimonic well-being to also associate with reduced CTRA gene expression. Five of the 6 major sub-domains of psychological well-being predicted reduced CTRA gene expression when analyzed separately, and 3 remained distinctively prognostic in mutually adjusted analyses. All associations were independent of demographic characteristics, health-related confounders, and RNA indicators of leukocyte subset distribution. These results identify specific sub-dimensions of eudaimonic well-being as promising targets for future interventions to mitigate CTRA gene expression, and provide no support for any independent favorable contribution from hedonic well-being.

## Introduction

Research in human social genomics has linked adverse social and psychological conditions to increased immune cell expression of a “conserved transcriptional response to adversity” (CTRA) characterized by up-regulated expression of pro-inflammatory genes (e.g., *IL1B*, *IL8*, *PTGS2*, *TNF*) and down-regulated expression of genes involved in Type I interferon innate antiviral responses (e.g., *IFI*-, *ISG*-, *MX*-, and *OAS*-family genes) and antibody synthesis (e.g., *IGJ*) [1–3]. Similar leukocyte transcriptome shifts have been observed in experimental animal models of social stress and threat [4–7] and may play a role in human social epidemiology because inflammation contributes to the development of multiple chronic illnesses [2, 8]. However, little is known about how positive psychological or social conditions (well-being) might affect gene expression. Such results may help illuminate the biological basis for the prospective health advantages associated with psychological well-being in epidemiologic studies [9, 10], and might potentially help guide the development of future interventions to reduce CTRA gene expression.

In an initial study of the transcriptomic correlates of positive psychological states, Fredrickson et al. [11] reported an association between reduced CTRA gene expression and scores in one domain of an established summary measure of well-being known as the Mental Health Continuum—Short Form (MHC-SF, also known as the Short Flourishing Scale) [12–15]. The MHC-SF assesses 2 broad domains of positive mental health or flourishing: hedonic well-being (high levels of positive emotion and life satisfaction) and eudaimonic well-being (psychological and social processes that transcend immediate self gratification and strive toward more deeply meaningful and pro-social goals) [15]. In the conceptual system underlying the MHC-SF, psychological well-being and social well-being comprise distinct but related sub-components of eudaimonia, resulting in a 3-d representation of human well-being (hedonic, psychological, and social well-being) [12–15]. Fredrickson et al. [11] found that a composite score of MHC-SF items tapping social and psychological dimensions of eudaimonic well-being was associated with down-regulated expression of the CTRA transcriptome profile in peripheral blood mononuclear cells (PBMC)

The initial results of Fredrickson et al. [11] generated substantive interest in defining the specific aspects of eudaimonic well-being that contribute to associations with gene expression [16–18]. One question regards whether eudaimonic well-being should be theoretically conceptualized as it is in the MHC-SF—as comprising both intrapsychic aspects of psychological well-being and outward-facing aspects of social well-being [12–15, 19, 20]. Other theorists have conceptualized eudaimonia predominately in terms of psychological well-being [17, 21–24], suggesting that it may be theoretically and psychometrically appropriate to consider psychological well-being separately from social well-being. The question then arises whether CTRA gene expression would continue to associate with measures of either psychological or social well-being, or both, when considered as separate dimensions. An alternative conceptual approach does not seek to distinguish among sub-domains of well-being but instead integrates measures of hedonic, psychological, and social well-being into a single overall categorical assessment of flourishing mental health [10, 15, 19]. Others have raised methodological questions about the initial results. Coyne [25] raised concerns regarding the possible collinearity of hedonic and eudaimonic well-being scores (given that these 2 domains are partially correlated). Brown and colleagues [18] questioned the integration of psychological and social well-being into a single integrated measure of eudaimonia, suggested the use of mixed effect linear models to address the issue of correlated residuals in pooled association analyses involving multiple CTRA indicator genes, and concluded that the initial results were “spurious” and “the chances of a successful reproduction … remote.”

The present analyses use data from 2 new independent study samples to assess the generalizability of CTRA associations with eudaimonic well-being and to identify the specific sub-domains of eudaimonic well-being that are most centrally involved in this relationship. Analyses also address potential collinearity among well-being measures and statistical inference in the presence of correlated residuals. Results from the confirmation study, a pooled analysis of the discovery and confirmation samples, and a separate generalization study using more robust measures of psychological well-being (Ryff Scales for Psychological Well-Being; Ryff-PWB) all find an inverse association between CTRA gene expression and eudaimonic well-being and converge in identifying psychological well-being as a key carrier of that relationship.

## Results

### Confirmation study

#### Sample characteristics and well-being

**Fig 1:**
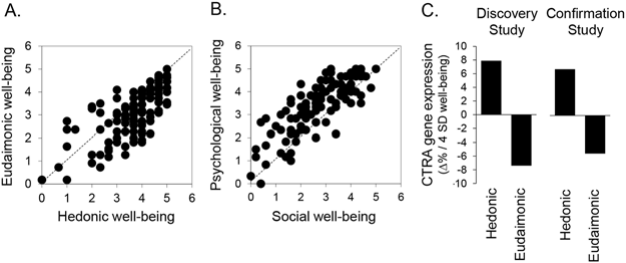
Well-being and CTRA gene expression. (A) Relationship between MHC-SF hedonic and eudaimonic well-being domain scores (2-d representation). (B) Relationship between psychological well-being and social well-being scores (the 2-d eudaimonic domain within the 3-d well-being representation). (C) Point estimates of average association coefficients relating range-spanning variations in hedonic and eudaimonic well-being scores [-2 SD, +2 SD] to unstandardized (log_2_ metric) gene expression values for 52 CTRA indicator genes (reverse scoring 34 inverse components) in the discovery study (n = 76) and confirmation study (n = 122). Log_2_ association coefficients are transformed to % difference in average CTRA transcript abundance to facilitate interpretation.

Characteristics of the confirmation sample are presented in Table 1 and reflect a middle-aged, predominately white sample of NC adults with a slight preponderance of women (60%) and generally healthy characteristics (mean BMI = 27 and low rates of illness symptoms and smoking). MHC-SF scales measuring hedonic and eudaimonic well-being showed good reliability (Table 1; all α >. 80). As in the discovery study [11], scores on hedonic and eudaimonic well-being scales were positively correlated (Fig. 1A, *r*(120) = +.74, *p* <. 0001) but also contained significant unshared variance (scale reliability α values exceeded the cross-scale *r* ^2^ value of. 55, and Confirmatory Factor Analysis [CFA] models hypothesizing 2 distinct eudaimonic and hedonic factors fit significantly better than did a model hypothesizing 1 common well-being factor, *X* ^2^(1) = 39.03, *p* <. 0001; S1 Table). Joint distributions showed one major component of roughly comparable scores on hedonic and eudaimonic well-being (Fig. 1A main diagonal) and a second major subpopulation with hedonic scores > eudaimonic scores (right-shifted from main diagonal). Only 22% of participants showed eudaimonic predominance (eudaimonic score ≥ hedonic score; *p* <. 0001 by sign test). In the conceptual model underpinning the MHC-SF, eudaimonic well-being is comprised of distinct dimensions of psychological well-being and social well-being [12, 15]. MHC-SF psychological and social well-being scales showed good reliability in this sample (Table 1) and scores on these two sub-scales were correlated (Fig. 1B, *r*(120) = +.80, *p* <. 0001). However, the scales also tapped significant unshared variance (α values > cross-scale *r* ^2^ =. 63, and CFA models specifying 3 distinct hedonic, psychological, and social well-being factors fit significantly better than did the 2-factor model specifying only general hedonic and eudaimonic factors, *X* ^2^(2) = 8.56, *p* =. 0138; S1 Table). An alternative 2-d scoring system advanced by Brown et al. [18] based on ad hoc factor analysis showed poorer fit than the standard MHC-SF 2-d model (S1 Table). However, good fit statistics were observed for an alternative 3-d variant proposed by Brown et al. that reallocates 2 social well-being items to the psychological well-being scale (comparison to the MHC-SF 2-d model, *X* ^2^(2) = 21.98, *p* <. 0001; S1 Table). Thus, gene expression analyses examined the original 2-d representation in which CTRA associations were initially identified [11] as well as both alternative 3-d representations of well-being.

**Table 1:** Confirmation Study Sample Characteristics.

A.	Mean (SD) or %	r (p-value) with Hedonic well-being^1^	r (p-value) with Eudaimonic well-being^1^	r (p-value) with Psychological well-being^1^	r (p-value) with Social well-being^1^	r (p-value) with Total MHC-SF well-being^1^	r (p-value) with categorical Flourishing^2^
Age (Years)	48.4 (8.8)	+.15 (.1036)	+.18 (.0426)	+.15 (.0919)	+.20 (.0277)	+.18 (.0420)	+.22 (.0135)
Sex (Female)	60.0%	+.03 (.7170)	+.11 (.2328)	+.13 (.1433)	+.07 (.5366)	+.10 (.2876)	+.06 (.5366)
Race/ethnicity^1^		+.13 (.5614)	+.09 (.8034)	+.05 (.9530)	+.13 (.5904)	+.09 (.8034)	n/a
White	76.1%						
Black	15.4%						
Hispanic	2.6%						
Asian	6.0%						
Body Mass Index (kg/m^2^)	27.4 (6.2)	+.04 (.6357)	+.02 (.8268)	+.03 (.7152)	+.00 (.9857)	+.02 (.7731)	+.04 (.6256)
Smoking history (yes/no)	3.3%	+.11 (.2467)	+.06 (.5090)	+.09 (.3110)	+.02 (.8634)	+.07 (.4203)	+.00 (.9999)
Alcohol history (yes/no)	74.6%	-.15 (.1056)	-.03 (.7086)	-.05 (.5967)	-.01 (.8845)	-.06 (.4998)	+.02 (.8369)
Illness symptoms (0–8 scale^3^)	1.05 (0.9)	-.15 (.0940)	-.12 (.1794)	-.09 (.3044)	-.14 (.1171)	-.13 (.1383)	-.13 (.1645)
Depressive symptoms (0–60 scale^4^)	12.5 (9.4)	-.53 (<. 0001)	-.52 (<. 0001)	-.53 (<. 0001)	-.44 (<. 0001)	-.54 (<. 0001)	-.35 (<. 0001)
**B.**		Hedonic well-being^5^	Eudaimonic well-being^5^	Psychological well-being^5^	Social well-being^5^	Total MHC-SF well-being^5^	Categorical Flourishing^2^
Scale mean (SD) or % prevalence		3.63 (1.08)	3.08 (1.06)	3.40 (1.11)	2.70 (1.14)	3.20 (1.02)	50%
Reliability (Cronbach α)		+.87	+.92	+.89	+.82	+.93	n/a

^1^. Simple (unadjusted) association with well-being: *r* point estimate or ANOVA effect size *r* (*p*-value).

^2^. Flourishing: ≥ 1 of 3 hedonic well-being signs and ≥ 6 of 11 eudaimonic well-being signs experienced “5–6 times a week” or “every day”.

^3^. Frequency of 13 minor illness symptoms over the past 2 weeks, scaled from 0 = not at all to 8 = very frequently.

^4^. CES-D

^5^. MHC-SF well-being scores scale from 0 = “never” to 5 = “every day”

#### CTRA gene expression

Table 2A reports results from mixed effect linear model analyses of the confirmation sample data (n = 122) relating average expression of 52 CTRA indicator genes to a 2-dimensional representation of well-being using continuously scored MHC-SF measures of hedonic and eudaimonic well-being as in the discovery study [11]. (One of the 53 CTRA indicator genes used in the discovery study was unavailable in the confirmation study primary data set—*IL6*). Analyses controlled for the same potential confounders as in the discovery study: age, sex, white vs. non-white race, BMI, smoking, alcohol consumption, illness symptoms, and abundance of transcripts marking the relative prevalence of CD4+ T cells, CD8+ T cells, B lymphocytes, NK cells, and monocytes within the PBMC pool. Results indicated a significant relationship between this 2-d representation of well-being and the CTRA indicator profile reflecting up-regulated expression of pro-inflammatory genes and down-regulated expression of genes involved in Type I interferon responses and antibody production (*F*(2, 104) = 14.19, *p* <. 0001). Analysis of domain-specific coefficients indicated a significant inverse association between eudaimonic scores and gene expression but no significant association with hedonic scores (Table 2A). Association coefficients were directionally interpretable because hedonic and eudaimonic domain scores were not substantially collinear (all variance inflation factor [VIF] values < 10; Table 2A). In direct comparison of standardized association coefficients, the magnitude of CTRA association with eudaimonic scores significantly exceeded that for hedonic scores (*t*(104) = -2.59, *p* =. 0109). Ancillary sensitivity analyses also found that CTRA associations with eudaimonic well-being were independent of depressive symptoms, did not vary as a function of alcohol consumption, and also emerged in a statistical model that specified a fully saturated (unstructured) covariance matrix (S1 File Supplemental Results and S2 and S3 Tables).

**Table 2:** Association of well-being with gene expression: Confirmation Study.

	Well-being dimension	Association *b* ± SE^1^	Test statistic	*p*-value	VIF^2^
**A. 2-dimensional**					
	Hedonic well-being	0.085 ± 0.121	*t*(104) = 0.70	.4829	2.46
	Eudaimonic well-being	-0.509 ± 0.125	*t*(104) = -4.08	<. 0001	2.59
**B. 3-dimensional**					
	Hedonic well-being	0.098 ± 0.122	*t*(103) = 0.80	.4243	2.49
	Psychological well-being	-0.384 ± 0.153	*t*(103) = -2.50	.0139	3.95
	Social well-being	-0.163 ± 0.137	*t*(103) = -1.19	.2370	3.14
**C. 1-dimensional**					
	Total well-being	-0.439 ± 0.086	*t*(105) = -5.11	<. 0001	1.17
**D. Categorical**					
	Flourishing mental health	-0.615 ± 0.176	*t*(105) = -3.49	.0007	1.17

^1^. Partial regression coefficients relating standardized gene expression values to standardized scores on 1-, 2-, and 3-d representations of well-being (A, B, C) or a categorical representation of flourishing mental health (D). All associations are adjusted for age, sex, race, BMI, smoking, alcohol consumption, illness symptoms, and gene transcript covariates marking major leukocyte subsets.

^2^. Variance Inflation Factor. Values > 10 indicate significant multicollinearity.

Similar results also emerged from pooled analyses of the combined discovery and confirmation data sets (n = 198; Table 3A). CTRA gene expression was significantly associated with scores in the 2-dimensional well-being space (*F*(2, 179) = 3.76, *p* =. 0251), and domain-specific parameters indicated a significant inverse association with eudaimonic scores but no significant association with hedonic scores (Table 3A). CTRA association with eudaimonic scores significantly exceeded that for hedonic scores in direct contrasts (*t*(179) = -2.40, *p* =. 0173), and ancillary analyses including interaction terms showed no significant difference in association magnitude across study samples (Study x Hedonic: F(1, 177) = 2.07, *p* =. 1518; Study x Eudaimonic: F(1, 177) = 0.85, *p* =. 3566). The 2 samples also showed similar magnitudes of association between unstandardized (log_2_) CTRA gene expression and well-being scores (Fig. 1C).

**Table 3:** Association of well-being with gene expression: pooled Discovery and Confirmation Studies.

	Well-being dimension	Association *b* ± SE^1^	Test statistic	*p*-value	VIF^2^
**A. 2-dimensional**					
	Hedonic well-being	0.074 ± 0.042	*t*(179) = 1.77	.0781	2.44
	Eudaimonic well-being	-0.116 ± 0.043	*t*(179) = -2.71	.0074	2.54
**B. 3-dimensional**					
	Hedonic well-being	0.059 ± 0.042	*t*(178) = 1.39	.1663	2.53
	Psychological well-being	0.015 ± 0.052	*t*(178) = 0.29	.7702	3.82
	Social well-being	-0.126 ± 0.045	*t*(178) = -2.81	.0055	2.83
**C. 1-dimensional**					
	Total well-being	-0.050 ± 0.028	*t*(180) = -1.78	.0775	1.07
**D. Categorical**					
	Flourishing mental health	-0.051 ± 0.056	*t*(180) = -0.91	.3637	1.06

^1^. Partial regression coefficients relating standardized gene expression values to standardized scores on 1-, 2-, and 3-d representations of well-being (A, B, C) or a categorical representation of flourishing mental health (D). All associations are adjusted for age, sex, race, BMI, smoking, alcohol consumption, illness symptoms, and gene transcript covariates marking major leukocyte subsets.

^2^. Variance Inflation Factor. Values > 10 indicate significant multicollinearity.

#### Alternative representations of well-being

Table 2B reports results from parallel mixed effect linear model analyses relating CTRA gene expression to a 3-d representation of well-being comprised of continuous MHC-SF scores assessing hedonic, psychological, and social well-being. Results again indicated a significant omnibus relationship between scores in the 3-d well-being space and the CTRA indicator profile (*F*(3, 103) = 9.64, *p* <. 0001). That association was carried by the 2-d eudaimonic domain comprised of psychological and social well-being scores (*F*(2, 103) = 8.66, *p* =. 0003; residual effect, *F*(1, 103) = 0.64, *p* =. 4243). Dimension-specific coefficients indicated a significant inverse association between psychological well-being and gene expression but no significant associations involving hedonic or social well-being (Table 2B). However, direct comparison analyses did not indicate a significant difference in the magnitude of inverse CTRA association with psychological vs. social well-being scores (*t*(103) = -0.85, *p* =. 3956), and separate analyses of psychological and social well-being scores in isolation from one another (and in isolation from hedonic well-being scores, but adjusted for all other covariates) showed inverse CTRA associations for both psychological well-being (*b* = -.446 ±. 086, *t*(105) = -5.20, *p* <. 0001) and social well-being (*b* = -.396 ±. 084, *t*(105) = -4.71, *p* <. 0001). Similar results also emerged using the alternative 3-d scoring system advanced by Brown et al. [18] (S1 File Supplemental Results and S4A Table). This suggests that the substantial variance shared in common by psychological and social well-being (64% of observed score variance) carried most of the association with gene expression.

In ancillary sensitivity analyses, CTRA associations with the 2-d eudaimonia domain were also independent of depressive symptoms, did not vary as a function of alcohol consumption, and consistently emerged in statistical models that specified an unstructured covariance matrix (S1 File Supplemental Results and S2 and S3 Tables).

Pooled sample analyses (Table 3B) yielded similar results indicating an overall association of scores in the 3-d well-being space with CTRA gene expression (*F*(3, 178) = 3.56, *p* =. 0154), with effects carried predominately by the 2-d eudaimonia domain (*F*(2, 178) = 5.25, *p* =. 0061; residual effect: *F*(1, 178) = 1.93, *p* =. 1663). Psychological and social well-being scores were strongly correlated in the pooled data (*r*(196) = +.79, *p* <. 0001), and dimension-specific coefficients identified a significant inverse CTRA association only for MHC-SF social well-being scores (Table 3B). However, direct comparison of social and psychological well-being score associations with CTRA gene expression showed no significant difference (*t*(178) = -1.64, *p* =. 1028). In analyses of each dimension separately, a significant inverse association was observed for social well-being scores (*b* = -.076 ± 0.028, *t*(180) = -2.73, *p* =. 0069), but not for psychological well-being scores (*b* = -0.036 ± 0.028, *t*(180) = -1.29, *p* =. 1970). Similar results again emerged using the alternative 3-d scoring system advanced by Brown et al. [18] (S1 File Supplemental Results and S4B Table).

Given the apparent role of shared variation among psychological and social well-being scores, we also examined CTRA gene expression associations with a 1-d score integrating across all 3 well-being dimensions (average over all MHC-SF items) [15] and a categorical representation of flourishing mental health based on all 3 MHC-SF well-being dimensions [15]. Confirmation study analyses showed a significant inverse association with the 1-d total well-being score (Table 2C), but that association only approached statistical significance in the pooled data set (Table 3C). Confirmation study analyses also showed significantly lower CTRA gene expression among those classified as showing flourishing mental health (Table 2D), but this association was not significant in the pooled sample (Table 3C). These results suggest that integration of hedonic score variance tends to degrade the strength and reliability of associations between MHC-SF eudaimonic well-being scores and CTRA gene expression.

### Generalization study

#### Sample characteristics and well-being

Parallel analyses were conducted on data from an independent archival data set (Gene Expression Omnibus GSE45329) that contained a more robust measure of eudaimonic well-being in the Ryff Scales for Psychological Well-Being (Ryff-PWB) [17, 21]. Specifically, whereas psychological well-being is assessed by 6 items in the MHC-SF, it is assessed by 54 items (6 scales of 9 items each) in the Ryff-PWB. Characteristics of the generalization study sample are presented in Table 4 and reflect a middle-aged, predominately white sample of Vancouver BC adults with a preponderance of women (79%) and generally healthy characteristics (mean BMI = 26 and low rates of smoking and alcohol consumption). Ryff-PWB scale scores measuring 6 major dimensions of eudaimonic well-being showed good reliability (Table 4) and, as in previous studies [17, 21], moderate levels of inter-correlation (*R* ^2^ ranging from. 14 to. 52, all *p* <. 01). Thus, gene expression analyses examined both a 6-d representation of psychological well-being and a 1-d total psychological well-being score that averaged across the 6 Ryff-PWB scales to capture their shared variance as an index of eudaimonia. No measures of hedonic well-being were available in this sample, and all participants were screened to be free of illness symptoms at the time of blood sampling.

**Table 4:** Generalization Study Sample Characteristics.

A.	Mean (SD) or %	*r* (*p*-value) with Purpose in life^1^	*r* (*p*-value) with Environmental mastery^1^	*r* (*p*-value) with Self-acceptance^1^	*r* (*p*-value) with Autonomy^1^	*r* (*p*-value) with Personal growth^1^	*r* (*p*-value) with Positive relations^1^
Age (Years)	45.3 (5.6)	+.05 (.5743)	+.20 (.0402)	+.18 (.0713)	+.01 (.9312)	+.08 (.4178)	+.16 (.1054)
Sex (Female)	79.4%	+.11 (.2572)	-.05 (.6077)	+.08 (.4270)	-.12 (.2291)	+.08 (.4157)	+.22 (.0211)
Race/ethnicity^1^		+.15 (.6797)	+.10 (.8963)	+.25 (.1686)	+.25 (.1689)	+.26 (.1298)	+.11 (.8704)
White	57.0%						
Chinese	18.7%						
Indian	7.5%						
Other Asian	12.2%						
Other Ethnicity	4.7%						
Body Mass Index (kg/m^2^)	25.8 (4.2)	-.07 (.5046)	+.00 (.9839)	+.06 (.5658)	+.19 (.0481)	+.05 (.6490)	-.12 (.2328)
Smoking history (yes/no)	5.6%	-.21 (.0264)	-.15 (.1150)	-.12 (.2030)	-.05 (.6122)	+.01 (.9203)	-.04 (.6669)
Alcohol history (yes/no)	31.8%	-.10 (.2950)	-.08 (.4126)	+.20 (.0390)	+.20 (.0414)	+.20 (.0410)	+.09 (.3832)
**B.**		Purpose in life^2^	Environmental mastery^2^	Self-acceptance^2^	Autonomy^2^	Personal growth^2^	Positive relations^2^
Scale mean (SD) or % prevalence		43.2 (6.2)	41.9 (6.5)	41.9 (7.3)	38.6 (7.2)	43.6 (6.8)	43.8 (7.7)
Reliability (Cronbach α)		+.67	+.78	+.82	+.81	+.82	+.83

^1^. Simple (unadjusted) association with psychological well-being: *r* point estimate or ANOVA effect size *r* (*p*-value).

^2^. Ryff-PWB item responses range from 1 = Strongly disagree to 6 = Strongly agree, yielding 9-item scale scores ranging from 9–54.

#### CTRA gene expression

Table 5 reports results from mixed effect linear model analyses of the generalization study data (n = 107) relating average expression of 53 CTRA indicator genes to eudaimonic well-being as measured by the Ryff-PWB. In analyses that controlled for age, sex, ethnicity, BMI, smoking, alcohol consumption, and leukocyte subset marker transcripts, results again indicated a significant inverse relationship between the 1-d total psychological well-being score and CTRA gene expression (Table 5A).

**Table 5:** Association of psychological well-being with gene expression: Generalization Study.

	Well-being dimension	Association *b* ± SE^1^	Test statistic	*p*-value	VIF^2^
**A. 1-dimensional**					
	Total psychological well-being	-0.0087 ± 0.0021	*t*(88) = -4.17	<. 0001	1.18
**B. 6-dimensional (independent)**					
	Purpose in life	-0.0071 ± 0.0021	*t*(88) = -3.40	.0010	1.18
	Environmental mastery	-0.0070 ± 0.0021	*t*(88) = -3.37	.0011	1.15
	Self-acceptance	-0.0112 ± 0.0021	*t*(88) = -5.26	<. 0001	1.29
	Autonomy	-0.0057 ± 0.0021	*t*(88) = -2.66	.0093	1.22
	Personal growth	0.0019 ± 0.0021	*t*(88) = 0.87	.3881	1.17
	Positive relations with others	-0.0061 ± 0.0021	*t*(88) = -2.85	.0055	1.21
**C. 6-dimensional (mutually adjusted)**					
	Purpose in life	-0.0045 ± 0.0024	*t*(83) = -1.85	.0681	1.92
	Environmental mastery	0.0026 ± 0.0028	*t*(83) = 0.90	.3704	2.66
	Self-acceptance	-0.0147 ± 0.0029	*t*(83) = -4.98	<. 0001	2.87
	Autonomy	-0.0082 ± 0.0021	*t*(83) = -3.89	.0002	1.46
	Personal growth	0.0159 ± 0.0025	*t*(83) = 6.31	<. 0001	2.10
	Positive relations with others	-0.0051 ± 0.0023	*t*(83) = -2.23	.0282	1.70

^1^. Partial regression coefficients relating centered log_2_ gene expression values to standardized scores on 1- or 6-d representations of psychological well-being. All associations are adjusted for age, sex, race, BMI, smoking, alcohol consumption, and gene transcript covariates marking major leukocyte subsets.

^2^. Variance Inflation Factor. Values > 10 indicate significant multicollinearity.

Analyses of the 6 individual Ryff-PWB scales considered in isolation yielded similar results (Table 5B), with significant inverse associations emerging for 5 of the 6 dimensions, including Purpose in Life, Environmental Mastery, Self-Acceptance, Autonomy, and Positive Relations with Others (all *p* <. 01). Personal Growth was the only dimension of psychological well-being that showed no significant association with CTRA gene expression.

In analyses of all 6 scales considered simultaneously (Table 5C), results again indicated a significant omnibus relationship between scores in the 6-d psychological well-being space and CTRA gene expression (*F*(6, 83) = 11.95, *p* <. 0001). Distinct statistically significant inverse associations emerged for the dimensions of Self-Acceptance, Autonomy, and Positive Relations with Others, and an inverse association for Purpose in Life approached statistical significance (*p* =. 0681). Personal Growth, which showed no significant CTRA association when analyzed in isolation from the other 5 dimensions, showed a significant positive CTRA association when analyzed in their context. The shifting significance of this association did not stem from multi-collinearity with the other dimensions of psychological well-being, however, as all VIF values again fell well below 10 (Table 5).

## Discussion

Analyses of 2 new independent study samples verified previous indications that eudaimonic well-being is associated with reduced CTRA gene expression [11] and identified psychological well-being as a primary carrier of that relationship. Gene expression associations with eudaimonic well-being were generalizable across distinct populations of healthy adults, robust to alternative statistical analysis by mixed effect linear modeling, not affected by any substantial degree of multicollinearity, and consistent across alternative conceptualizations of well-being as implemented in the MHC-SF and the Ryff-PWB scales. When well-being was measured by the MHC-SF, CTRA gene expression was inversely associated with the 1-d eudaimonia score within the 2-d well-being representation, and with the joint effects of psychological and social well-being (the 2-d eudaimonia domain) within the 3-d well-being representation. CTRA associations with MHC-SF 2-d eudaimonia domains emerged regardless of whether their 3-d scoring systems were theoretically derived [12–15] or empirically motivated [18]. In contrast, MHC-SF hedonic well-being showed no association with CTRA gene expression independent of its correlation with eudaimonia, and total well-being measures that integrated hedonic and eudaimonic measures showed weaker and less consistent associations with gene expression profiles than did distinct measures of eudaimonia. When eudaimonic well-being was measured using the Ryff-PWB scales, CTRA gene expression was inversely associated with both a 6-d representation of eudaimonia and a 1-d total score capturing their shared variance [17, 21]. Five specific dimensions of psychological well-being involved in this relationship included Purpose in Life, Environmental Mastery, Self-Acceptance, Autonomy, and Positive Relations with Others. The present findings thus converge with previous results [11] in identifying eudaimonic well-being as the primary source of associations between overall well-being and CTRA gene expression and provide no support for any independent favorable contribution from hedonic well-being. The consistency and robustness of these findings across 3 independent study samples also refutes claims that the initial discovery study findings [11] were somehow spurious or unreplicable [18, 25].

Psychometric analyses of confirmation study data also replicated previous findings [13–15, 26] that hedonic and eudaimonic well-being constitute correlated but empirically separable domains of general well-being. CFAs confirmed the presence of significant unique variance in hedonic and eudaimonic well-being scores in the 2-d representation of well-being, and CTRA gene expression showed significant differential associations with these 2 domains in both the confirmation study and the pooled analysis of discovery and confirmation data sets. Hedonic and eudaimonic well-being measures were correlated, but not so strongly as to induce material multicollinearity (all VIFs fell well below established thresholds) [27, 28]. Moreover, any collinearity that did exist could not account for the observed significant associations because collinearity acts to increase the sampling variability of estimated associations with gene expression and thereby reduces statistical power to detect significant relationships [27]. (Association estimates from linear statistical models remain unbiased and *p*-values remain accurate in the presence of correlated predictors [27], so the emergence of statistically significant associations in this study cannot be attributed to any effect of correlated regressors as suggested by Coyne [25]). The consistent emergence of differential CTRA associations with eudaimonic vs. hedonic well-being measures also indicates a clear empirical distinction between these two domains, and should motivate further research on the biological correlates of distinct forms of experienced well-being [17, 20, 29–32].

Psychometric analyses of the MHC-SF also supported the sub-division of general eudaimonic well-being into distinct dimensions of psychological and social well-being (i.e., a 3-d representation of well-being encompassing a 2-d eudaimonia domain) [13–15, 26, 33–35]. This 3-d representation allows comparison of biological associations obtained under Keyes’ theoretical conception of eudaimonia (as comprising both social and psychological well-being) with those arising from other theoretical perspectives such as Ryff’s that conceptualize eudaimonia predominately in terms of psychological well-being [17, 21–24]. Consistent with both perspectives, 2-d eudaimonia domain scores within the 3-d well-being representation showed consistent associations with CTRA gene expression. Confirmation sample analyses also supported the more focal conceptualization of eudaimonia [17, 21–24] in finding CTRA gene expression to associate with psychological well-being (both when considered in isolation from social and hedonic well-being and when controlling for them). However, in analyses of the pooled discovery and confirmation study data, psychological well-being scores did not significantly associate with CTRA gene expression whereas social well-being scores did (both in the absence of psychological and hedonic well-being and when controlling for them). The instability of these sub-dimension-specific results for the MHC-SF across data sets suggests that the primary carrier of well-being’s association with gene expression is the >60% of variance shared in common by psychological and social well-being (with sampling variability in their minor unshared variance determining which dimension emerged as most prognostic in a given data set) [36]. A key role for shared variance is also consistent with the fact that score variation within the overall MHC-SF 2-d eudaimonia space showed consistently significant associations with gene expression in both the confirmation study and pooled analyses. If the shared variance in psychological and social well-being represents the primary carrier of CTRA associations, it may not make conceptual sense to examine these two dimensions separately as distinct predictors of biology even if it is statistically and psychometrically feasible to do so. Shared variance would best be captured in the MHC-SF by integrating social and psychological well-being measures into a 1-d eudaimonia domain score within the 2-d well-being representation (as in [11]). Indeed, that integrated measure showed the most stable and robust associations with gene expression in the confirmation study. This approach is also consistent with findings from the generalization study, in which more robust and detailed measures of eudaimonia were available in the Ryff-PWB scales [17, 37] and results again found robust CTRA associations with the overall 6-d eudaimonia space as well as with the 1-d summary score. Generalization study results also indicated a key role for shared variance among the 6 dimensions of psychological well-being in finding highly significant inverse associations with CTRA gene expression for 5 of the 6 dimensions when considered in isolation from one another, but for only 3 dimensions when considered simultaneously (i.e., partialling out shared variance).

The present confirmation study results differ from those of the discovery study in finding no consistent association of hedonic well-being with up-regulated CTRA gene expression (i.e., independent of eudaimonic well-being). However, some ancillary analyses did indicate positive CTRA associations with hedonic well-being in a subset of confirmation study participants (alcohol consumers) and across all study participants in one specific model estimated using an unstructured covariance matrix (S2 Table row J). No measures of hedonic well-being were available in the generalization study, so future research will be required to determine whether there exists any general or context-specific adverse CTRA association with hedonic well-being independent of eudaimonic well-being. What the discovery and confirmation studies both show most consistently, however, is that CTRA gene expression is more strongly associated with an individual’s relative balance of eudaimonic vs. hedonic well-being (i.e. the “eudaimonic predominance” difference: eudaimonic well-being—hedonic, the test of which equates to the reported differences in association coefficients for eudaimonic vs. hedonic well-being) than it is with absolute levels of either type of well-being considered in isolation.

The present results contradict assertions by Brown et al. [18] and Coyne [25] that the discovery study results initially reported in Fredrickson et al. [11] were spurious and unreplicable. Although Brown, Coyne, and colleagues judged the original findings to be “no more than a product of chance” and “the chances of a successful reproduction … to be remote,” the present results show that CTRA associations with eudaimonic well-being are indeed replicable across 3 independent study samples, robust to alternative psychometric representations of eudaimonia (including alternative measurement by MHC-SF and Ryff-PWB scales), not affected by multicollinearity, and readily observed in the context of mixed effect linear models that account for correlations in the expression of the 53 CTRA indicator genes. How could Brown, Coyne, and colleagues have arrived at conclusions at such variance with empirical data? Each of their analyses involved fatal statistical flaws that ultimately undermined the validity of their conclusions. Coyne [25] noted that hedonic and eudaimonic well-being were empirically correlated, but his further extrapolation that they were substantively collinear (i.e., contained no significant unique variance) is empirically false [38] (which is verified by the present CFA results showing significant unique variance in MHC-SF 2-d and 3-d representations of well-being as well as by low VIF values). Brown et al. [15] conducted factor analyses and a “bitmap” systematic combinatorial re-scoring of the discovery study data to argue that alternative scorings of the MHC-SF might yield more reliable associations with gene expression than did the summary score aggregating over items that Keyes defined on theoretical grounds as tapping eudaimonic well-being. They also inferred from their bitmap analysis that the pooled association estimator used in the discovery study generated spurious false positive results. However, subsequent analysis of Brown’s bitmap procedure identified fundamental errors of statistical logic that invalidated all of its estimates of analytic error [39, 40]. The bitmap procedure also failed to account for sampling variability and thus systematically capitalized on chance to yield unreplicable psychometric findings [39, 40]. Capitalization on chance also plagued the exploratory and confirmatory factor analyses used by Brown et al. in an attempt to re-allocate MHC-SF items to alternative factor structures derived de novo from the discovery study data. Based on established statistical guidelines [41, 42], the limited sample size available in the discovery study is sufficient to estimate aggregate level properties such as the number of factors present or the fit of an a priori-specified factor structure, but is insufficient for the more statistically demanding task of de novo factor discovery and reliable re-allocation of items to those ad hoc factors. Brown et al.’s capitalization on chance variations in the discovery data set explains why their 2-d MHC-SF factor solution failed to replicate in the present confirmation study whereas replicable findings readily emerged for eudaimonia domain scores that were derived from more robust factor analyses of MHC-SF structure in multiple previous large psychometric studies [15]. Given that the conclusions of Brown et al. derived entirely from their application of ad hoc factor discovery in an insufficiently powered sample and an invalid bitmap analysis, it is unsurprising that their conclusions find little empirical support in the 2 new data sets analyzed here.

Limitations of the present studies include their cross-sectional designs, which preclude conclusions about the causal direction of the observed associations, demographically homogenous study samples, which limits generalization to other populations, and the lack of measures of immunologic function and health outcomes to gauge the medical significance of the observed transcriptome differences. These analyses controlled for potential confounding by major demographic, health, and behavioral risk factors, as well as by variations in leukocyte subset prevalence, but other joint influences may remain unmeasured and potentially affect the magnitude of association estimates reported here. This study confirms associations of well-being with the biological theory-derived CTRA gene set [3, 11], but these analyses do not address the possibility that other major dimensions of the leukocyte transcriptome might also be found to associate with well-being in future research (e.g., the empirically-derived blood-informative transcript sets representing major axes of variation in the leukocyte transcriptome [43]). Future research is also needed to extend these findings to larger and more diverse samples, experimentally manipulate well-being to assess its possible causal effects on gene expression, and identify biological pathways that may mediate relationships between experienced well-being and leukocyte gene expression profiles.

Despite these limitations, the present results do advance our understanding of the relationship between positive psychological states and gene expression by identifying eudaimonic well-being as a key psychological carrier of such relationships, and further defining central roles for 5 major sub-dimensions of eudaimonic well-being as conceptualized in Ryff’s measures of psychological well-being [17, 37]. These results also clarify distinctive roles of eudaimonic and hedonic well-being in finding no support for any favorable CTRA association with hedonic well-being. The present findings may help illuminate the molecular basis for the prospective health advantages associated with well-being in epidemiologic studies and potentially inform the development of new interventions to promote such effects.

## Materials and Methods

### Confirmation Study

#### Procedure

To assess the replicability of previous results [11], we performed direct replication analyses on data from an independent confirmation sample of 176 adults recruited approximately 1 yr later from the Durham and Orange County regions of NC. Participants were healthy 35–64 yr-old English-speaking adults who provided written informed consent, completed online questionnaires assessing demographic characteristics and well-being (MHC-SF), and then attended a late afternoon laboratory session in which they were asked to provide a 20 ml venipuncture blood sample under resting conditions and were assessed for height and weight. The laboratory visit served as the baseline for a subsequent randomized controlled intervention study examining the effects of different meditation practices on psychological and biological outcomes (to be reported elsewhere). All intervention procedures (including randomization) occurred after collection of baseline data analyzed here, and participants were informed about the study topic prior to enrollment. All procedures were approved by the Institutional Review Board of the University of North Carolina at Chapel Hill. Additional procedural details are available in S2 File Supplemental Materials and Methods.

#### Measures

Age, sex, race/ethnicity, smoking history, alcohol consumption, 2-week history of 13 minor illness symptoms (e.g., headache, upset stomach), and depressive symptoms were assessed by self-report as in [11]. MHC-SF scales were scored according to established procedures [15] to generate 4 alternative representations of well-being: (1.) a 3-d representation involving hedonic, psychological, and social dimensions, (2.) a 2-d representation involving hedonic and eudaimonic domains (the later pooling psychological and social well-being items), (3.) a 1-d representation integrating over all MHC-SF items, and, (4.) a categorical representation of flourishing mental health defined by reporting ≥ 1 of 3 hedonic signs and ≥ 6 of 11 eudaimonic signs experienced “every day” or “5–6 times a week.” Additional measurement details are provided in S2 File Supplemental Materials and Methods.

#### CTRA gene expression

Genome-wide transcriptional profiling was conducted on PBMC from all 166 participants who agreed to provide blood samples, utilizing the same Illumina HT-12 v4 BeadArray assay and analysis procedures as in [11]. Additional assay details are available in SI, and confirmation study data are posted as Gene Expression Omnibus GSE55762. Random variations in Illumina BeadArray production resulted in missing *IL6* determinations for some samples [44], so we conservatively deleted all *IL6* data (rather than attempting to impute missing values), resulting in a 52-gene CTRA score in the confirmation study.

#### Statistical analyses

Statistical analyses were conducted using SAS v9.3 (SAS Institute, Cary NC). Associations among variables were assessed by Pearson correlation, point-biserial correlation, one-way analysis of variance (ANOVA), and *X* ^2^ tests, depending on the nature of the variables assessed. Potential multicollinearity was quantified by variance inflation factors (VIF = [1-*R* ^2^]^-1^ from a multiple regression relating each regressor to all others), with comparison to the established threshold value of 10 [27, 28]. Cronbach’s α assessed reliability of MHC-SF scales in this sample, and confirmatory factor analyses (CFA) tested the presence of reliable unique variance in each scale by comparing the fit of models specifying (1.) a single common well-being factor, (2.) a 2-d factor structure involving hedonic and eudaimonic domains (i.e., pooling psychological and social well-being items), and (3.) a 3-d factor structure involving dimensions of hedonic, psychological, and social well-being [13–15, 26, 33–35]. Reliability of alternative scoring approaches advanced in [18] was also examined. CFA models were fit as detailed in S2 File Supplemental Materials and Methods.

Gene expression data were analyzed using mixed effect linear models [45] treating the 52 CTRA indicator transcripts as repeated measures tested for average association with well-being scores. All associations were adjusted for potential confounding by the same set of a priori-selected covariates as in [11]: age, sex, white vs. non-white race/ethnicity, BMI, alcohol consumption, smoking, current illness symptoms, and 8 RNA transcripts indicting the relative prevalence of CD4+ and CD8+ T lymphocytes, B lymphocytes, NK cells, and monocytes. Quantile-normalized gene expression data were log_2_-transformed and standardized within gene to mean = 0 and SD = 1, sign-inverted for negative elements of the CTRA gene set (31 interferon-related genes and 3 antibody-related genes) [11], and analyzed using SAS PROC MIXED with maximum likelihood estimation, a fixed repeated measures effect capturing systematic differences across the 52 CTRA indicator genes, and a heterogeneous compound symmetry covariance structure accounting for correlated residuals across the 52 CTRA indicator genes. Ancillary sensitivity analyses examined the effects of alternative model specifications including unstructured covariance matrices, control for depressive symptoms, and interaction terms specifying differing CTRA associations as a function of alcohol consumption. Missing demographic or behavioral data (n = 44) and invalid RNA assay results (n = 1, who was also among those missing behavioral data) reduced the analyzed gene expression data set to 122 participants. In addition to replication analyses in the confirmation study sample, we also conducted pooled analyses of the combined discovery and confirmation samples using the analysis model specified above with an additional covariate adjusting for systematic differences across studies. Point estimates of unstandardized (log_2_ metric) effect sizes were also computed as previously described [11] to facilitate biological interpretation. Additional analytic details are provided in S2 File Supplemental Materials and Methods.

### Generalization Study

#### Procedure

To assess the generalizability of findings from the discovery and confirmation studies, we performed additional conceptual replication analyses on archival data from 107 healthy adults recruited from the Vancouver BC metropolitan area (Gene Expression Omnibus series GSE45329). Data come from healthy 34–61 yr-old English-speaking adults who provided written informed consent and attended a laboratory visit in which they completed measures of demographic characteristics, health-related behavior, and psychological well-being, were assessed for height and weight, and provided venipuncture blood samples for genome-wide transcriptional profiling. All procedures were approved by the Research Ethics Board of the University of British Columbia.

#### Measures

Age, sex, race/ethnicity, smoking history, and alcohol consumption were assessed by self-report, and participants completed 9-item versions of the 6 Ryff-PWB scales which were scored according to established procedures [21]. Analyses also examined a total PWB score averaging over the 6 scale scores.

#### CTRA gene expression

Genome-wide transcriptional profiling was conducted on PBMC utilizing the same Illumina HT-12 BeadArray assay and analysis procedures as in the confirmation study.

#### Statistical analyses

Statistical analyses utilized data from all 107 participants who had full gene expression, covariate, and Ryff-PWB data in GSE45329. Mixed effect linear model analyses relating Ryff-PWB scale scores to CTRA gene expression were conducted as described above for the confirmation study with the following differences: 1.) valid data were available for all CTRA indicator genes used in the discovery study [11] so analyses involved 53 CTRA transcripts; 2.) self-reported ethnicities were more heterogeneous than in the discovery and confirmation samples, so analyses controlled for a 5-category representation of ethnic groups showing > 5% prevalence (White, Chinese, Indian, Other Asian, Other ethnicity); 3.) analyses did not control for current illness symptoms because all participants were screened to be illness-free at the time of study participation; 4.) PROC MIXED analyses utilized an unstructured covariance matrix (heterogeneous compound symmetry specifications failed to converge and yielded unstable parameter estimates), and; 5.) gene expression values were standardized to mean = 0 but retained their native log_2_ dispersion (additional standardization to SD = 1 was not required to facilitate convergence of estimation algorithms but did result in unstable parameter estimates and reduced signal-to-noise ratios).

## Supporting Information

S1 Dataset Confirmation study MHC-SF item data. (XLSX)

S1 File Supplemental Results. (DOC)

S2 File Supplemental Materials and Methods. (DOC)

S1 Table Confirmatory Factor Analysis of alternative MHC-SF representations of well-being. (DOC)

S2 Table Confirmation study association of well-being with gene expression controlling for depressive symptoms. (DOC)

S3 Table Confirmation study association of well-being with gene expression: unstructured covariance matrix. (DOC)

S4 Table Association of alternative MHC-SF well-being measures with gene expression. (DOC)

S5 Table Confirmation study association of well-being with gene expression: Including imputed *IL6* data. (DOC)
