# Correction: Psychological Well-Being and the Human Conserved Transcriptional Response to Adversity

**DOI:** 10.1371/journal.pone.0157116

**Published:** 2016-06-03

**Authors:** Barbara L. Fredrickson, Karen M. Grewen, Sara B. Algoe, Ann M. Firestine, Jesusa M. G. Arevalo, Jeffrey Ma, Steve W. Cole

Following publication, the authors learned that the “Discovery Study” section of the originally published version of Fig 1C was derived from data that included a coding error in one control variable for one participant. This Correction includes a corrected version of Fig 1 with corrected values for the left 2 bars in Fig 1C being: Hedonic: + 3.6% and Eudaimonic: -7.2%. This is consistent with the direction of effects in the Discovery study reported in the original publication, although the magnitude of the effect for the hedonic association is attenuated. Both Discovery and Confirmation studies continue to show a positive association with gene expression for hedonic well-being, a negative association with gene expression for eudaimonic well-being, and a more favorable (negative) association of gene expression with eudaimonic well-being than with hedonic well-being.

**Fig 1:**
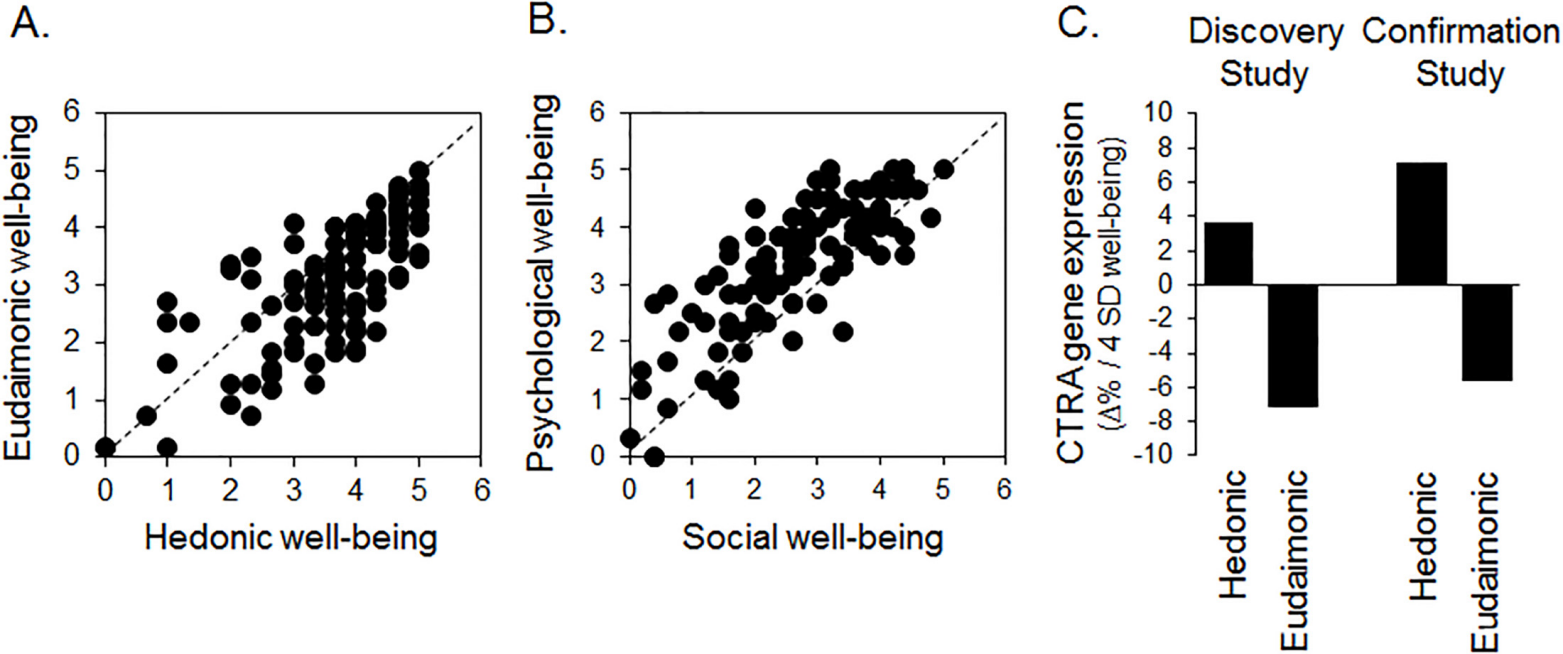
Well-being and CTRA gene expression. (A) Relationship between MHC-SF hedonic and eudaimonic well-being domain scores (2-d representation). (B) Relationship between psychological well-being and social well-being scores (the 2-d eudaimonic domain within the 3-d well-being representation). (C) Point estimates of average association coefficients relating range-spanning variations in hedonic and eudaimonic well-being scores [-2 SD, +2 SD] to unstandardized (log_2_ metric) gene expression values for 52 CTRA indicator genes (reverse scoring 34 inverse components) in the discovery study (n = 76) and confirmation study (n = 122). Log_2_ association coefficients are transformed to % difference in average CTRA transcript abundance to facilitate interpretation.

The discrepancy stemmed from use of the originally released version of the Discovery Study data set GEO GSE45330 for production of the original Fig 1C. The original version of GSE45330 was released in July 2013 and included a single mis-coded observation in which a value of “4” was included for what should have been a value of “0” indicating non-white race for study participant SOBC1_1299. The GSE45330 dataset was updated in July 2014 to correct this coding error. The corrected version of GSE45330 was used for the production of all other results in this article that utilized the Discovery Study data (including the findings reported in Table 3 that involve pooled analysis of the Discovery and Confirmation Studies). However, the original, uncorrected version of GSE45330 was inadvertently used in the production of the originally published Fig 1C.

The statistical analysis syntax used in the production of the corrected Fig 1C is also included in this Correction as S3 File.

## Supporting Information

S3 File Fig 1C Statistical Syntax. (PDF)
