# Supplementary material for: Psychological Well-Being and the Human Conserved Transcriptional Response to Adversity
Source: PLoS One. 2015 Mar 26;10(3):e0121839. doi: 10.1371/journal.pone.0121839 (PMC4374902; doi:10.1371/journal.pone.0121839)
Supplement: S1 File — (DOC) [file pone.0121839.s002.doc]

**Supplemental Results**

**Sensitivity analyses**

Ancillary analyses that controlled for depressive symptoms as measured by the Center for Epidemiologic Studies Depression scale [1] also continued to find significant inverse associations between eudaimonic well-being and CTRA gene expression (S2 Table).

Given the relatively high prevalence of alcohol consumption in the confirmation study sample, interaction analyses tested for potential differences in the magnitude of CTRA association with eudaimonia in participants with high vs. low levels of alcohol consumption. No significant interactions emerged for the 1-d measure of eudaimonic well-being within the 2-d well-being representation (*p* = .3940) or for the 2-d eudaimonia space of psychological well-being and social well-being within the 3-d well-being representation (*p* = .5109 and .1597, respectively). However, significant interactions did emerge for hedonic well-being in both 2- and 3-d well-being representations (both *p* < .001): hedonic well-being associated with down-regulated CTRA gene expression in the context of low alcohol consumption and up-regulated CTRA gene expression in the context of high alcohol consumption. Similar interactions emerged for total well-being representations that integrated over hedonic and eudaimonic well-being, with both 1-d total well-being scores and categorical flourishing mental health showing significant inverse associations with CTRA gene expression in low alcohol consumers but non-significant associations in high alcohol consumers (both interactions, *p* < .05).

To determine how the specified covariance structure for the 52 CTRA indicator genes might affect results, models were re-estimated using fully parameterized (unstructured) **R** matrices. Across unstructured covariance models fit by the 3 available estimation algorithms in SAS PROC MIXED (maximum likelihood, restricted maximum likelihood, and minimum variance quadratic unbiased estimation) [2], 3-d well-being representations consistently showed significant associations between the 2-d eudaimonia space and CTRA gene expression (all *F*(2,103) > 5.00, *p* < .01). In all 3 cases, dimension-specific parameters confirmed an inverse CTRA association with psychological well-being (all *p* < .01; S3 Table rows B, F, and J). All 3 unstructured covariance models also indicated significant positive associations of social well-being with CTRA gene expression that were not observed in primary analyses (all *p* < .01; S3 Table rows B, F, and J). One of the 3 analyses also indicated a positive association of hedonic well-being with CTRA gene expression (S3 Table row J). In analyses of the 2-d well-being representation, one estimation algorithm failed to converge (S3 Table row E), a second confirmed a significant inverse association between eudaimonic well-being and gene expression (S3 Table row I), and a third indicated no significant association (S3 Table row A). In analyses of categorical and 1-d representations of total well-being, the 2 estimation algorithms that yielded results both indicated inverse CTRA associations with the 1-d total well-being representation (both *p* < .05; S3 Table rows C and K) and neither indicated any significant association for the categorical representation of flourishing mental health (both *p* > .40; S3 Table rows D and L). Given the technical difficulties involved in estimating the 1,378 parameters required for the unstructured variance-covariance matrix specification (resulting in failure to converge, unstable parameter estimates, and inconsistent substantive findings across estimation algorithms), these unstructured covariance analyses of the confirmation study data should be interpreted with great caution.

**Alternative representations of well-being**

In addition to examining multi-dimensional well-being representations based on the theoretical background underpinning the MHC-SF [3, 4] and previous empirical analyses of its dimensional structure [5-10], we also examined alternative 2- and 3-d scoring systems advanced by Brown et al. [11] based on ad hoc factor analyses. Their alternative 2-d model hypothesizing one general well-being factor and a distinct social well-being dimension showed poor fit relative to the standard MHC-SF 2-d model hypothesizing distinct hedonic and eudaimonic item domains (S1 Table). However, good fit statistics were observed for the 3-d variant hypothesized by Brown et al. [11], which involves similar hedonic, social, and psychological well-being dimensions but reallocates 2 items from the established MHC-SF social well-being scale (how often does a respondent feel “that you had something to contribute to society” and “that you belonged to a community/social group”) to the psychological well-being scale (comparison to the MHC-SF 2-d model, *X*^2^(2) = 21.98, *p* < .0001; S1 Table). This alternative 3-d structure showed better overall model fit statistics than did the standard MHC-SF 3-d structure (S1 Table), but it yielded a social well-being scale with somewhat lower reliability than that of the established MHCF-SF social well-being scale (α = .76 vs. .82). The alternative psychological well-being scale showed acceptable reliability (α= .91). Reduced reliability of the alternative social well-being scale and stable or increased reliability of the alternative psychological well-being scale is to be expected based on changes in the number of items allocated to each.

Analyses relating CTRA gene expression to the alternative 3-d scoring system showed results similar to those observed using the established MHC-SF 3-d scoring system. Analyses of the confirmation study data showed significant CTRA association with the 3-d well-being space (*F*(3, 103) = 9.82, *p* < .0001), and that association was carried by the 2-d eudaimonia domain comprised of alternative psychological and social well-being dimensions (*F*(2, 103) = 8.97, *p* = .0003; residual *F*(1, 103) = 0.23, *p* = .6314). Dimension-specific analyses showed significant or near-significant associations for both psychological and social well-being scales in the 3-d context (S4 Table). Direct comparisons showed no difference in the magnitude of CTRA association for alternative psychological vs. social well-being scores (*t*(103) = -0.06, *p* = .9548) and analyses of these 2 dimensions in isolation from each other and from hedonic well-being showed similarly strong CTRA associations for each (alternative psychological well-being: *b* = -0.422 ± 0.086, *t*(105) = -4.89, *p* < .0001; alternative social well-being: *b* = -0.417 ± 0.083, *t*(105) = -5.03, *p* < .0001).

Analyses of the pooled discovery and confirmation study data again identified significant CTRA association with the overall 3-d well-being space (*F*(3, 178) = 6.42, *p* = .0004), with effects carried by the 2-d eudaimonia domain, *F*(2, 178) = 9.52, *p* = .0001; residual *F*(1, 178) = 0.55, *p* = .4589). However, significant dimension-specific associations emerged only for the alternative social well-being score (both in models controlling for hedonic and alternative psychological well-being scores, S4 Table, and in isolation, *b* = -0.111 ± 0.028, *t*(180) = -4.04, *p* < .0001). Alternative psychological well-being scores showed no CTRA association in the pooled sample (either controlling for hedonic and alternative social well-being, S4 Table, or in isolation, *b* = -0.029 ± 0.028, *t*(180) = -1.04, *p* = .2992). Given substantial correlation among alternative psychological and social well-being scores (confirmation study *r*(120) = +.67; pooled *r*(196) = +.61, both *p* < .0001), the inconsistency in dimension-specific CTRA association estimates across samples again suggests a central role for variance shared between these two alternatively scored scales.

Collectively, these results confirm that CTRA associations with the overall 2-d eudaimonic item domain of the MHC-SF are robust to alternative scoring approaches and emerge consistently regardless of whether the “contribution” and “belonging” items are allocated to the social well-being score as theoretically derived [3, 4] or to the psychological well-being score as empirically suggested (S1 Table and [11]).

**References**
