# Supplementary material for: Psychological Well-Being and the Human Conserved Transcriptional Response to Adversity
Source: PLoS One. 2015 Mar 26;10(3):e0121839. doi: 10.1371/journal.pone.0121839 (PMC4374902; doi:10.1371/journal.pone.0121839)
Supplement: S2 File — (DOC) [file pone.0121839.s003.doc]

**Supplemental Materials and Methods**

**Confirmation Study Procedure**

To assess the replicability of previously reported associations between well-being and gene expression [1], we performed direct replication analyses on data from an independent confirmation sample of 176 adults recruited approximately 1 yr later from the Durham and Orange County regions of NC. Participants were healthy 35-64 yr-old English-speaking adults who provided written informed consent, completed online questionnaires assessing demographic characteristics and well-being (MHC-SF), and then attended a late afternoon laboratory session in which they were asked to provide a 20 ml venipuncture blood sample under resting conditions and were assessed for height and weight. The laboratory visit served as the baseline for a subsequent randomized controlled intervention study examining the effects of different meditation practices on psychological and biological outcomes (to be reported elsewhere). All intervention procedures including randomization occurred after collection of baseline data analyzed here, and participants were informed about the study topic prior to enrollment. All procedures were approved by the Institutional Review Board of the University of North Carolina at Chapel Hill.

**Measures**

Age, sex, race/ethnicity, smoking history, alcohol consumption, 2-week history of 13 minor illness symptoms (e.g., headache, upset stomach), and depressive symptoms were assessed by self-report as in [1]. BMI was computed based on research staff measurements of height and weight. Race was coded into general white/non-white categories for primary analyses because there were insufficient numbers of any individual non-white race/ethnicity to support reliable statistical estimation of group-specific deviations from the non-white category (Table 1). Alcohol and smoking history were assessed retrospectively 6-12 mo after baseline, due to the inadvertent omission of these items from the baseline survey. (Time scope of retrospective assessments covered the baseline period, no participant indicated a change in either exposure history during the past year, and sensitivity analyses omitting smoking and alcohol history covariates from gene expression analyses yielded substantively identical results.) Depressive symptoms were measured by the Center for Epidemiologic Studies Depression Scale [2]. Well-being was assessed by the Mental Health Continuum – Short Form (MHC-SF) [3], which is a 14-item self-report instrument assessing 3 dimensions of experienced well-being: (1.) hedonic well-being, as indexed by 3 items assessing how often respondents reported feeling “happy,” “interested in life,” and “satisfied with life,” (2.) social well-being, as indexed by 5 items assessing how often respondents reported feeling “that you had something to contribute to society,” “that you belonged to a community/social group,” “that our society is becoming a better place for people,” “that people are basically good,” “that the way our society works makes sense to you,” and, (3.) psychological well-being, as indexed by 6 items assessing how often respondents reported feeling “that you like most parts of your personality,” “good at managing the responsibilities of your daily life,” “that you had warm and trusting relationships with others,” “that you have experiences that challenge you to grow and become a better person,” “confident to think or express your own ideas and opinions,” and “that your life has a sense of direction or meaning to it.” Participants were asked, “In the past week, how often did you feel…..” each of the above, and they responded on a scale of 0 = “Never,” 1 = “Once,” 2 = “Twice,” 3 = “3 or 4 times a week,” 4 = “5-6 times a week,” and 5 = “Every day.” The 3 basic MHC-SF scales were scored according to established procedures [3] to generate 4 alternative representations of well-being: (1.) a 3-d representation involving hedonic, psychological, and social dimensions, (2.) a 2-d representation comprising hedonic well-being and a broader eudaimonic domain score pooling over psychological and social well-being items, (3.) a 1-d score integrating over all MHC-SF items, and, (4.) a categorical representation of flourishing mental health defined by reporting ≥ 1 of 3 hedonic signs and ≥ 6 of 11 eudaimonic signs experienced “every day” or “5-6 times a week.” All continuous scale scores (representations 1-3) showed acceptable reliability in this sample as indicated by Cronbach α coefficients > .80 (Table 1B).

**CTRA gene expression**

Genome-wide transcriptional profiling was conducted on PBMC from all 166 participants who agreed to provide blood samples, utilizing the same Illumina HT-12 v4 BeadArray assay procedures as in [1]. Briefly, PBMC were isolated from 20 ml venipuncture blood samples by ficol density gradient centrifugation. Cell pellets were lysed and stabilized in RNA extraction buffer RLT (Qiagen, Valencia CA) and frozen at -80^o^C prior to batch processing of the entire study sample. Sample processing involved extraction of total RNA using an automated nucleic acid processor (Qiagen RNEasy protocols performed on a QIAcube instrument according the manufacturer’s standard protocol), quantification of RNA mass and density (Nanodrop ND-1000), quantification of RNA integrity (Agilent Bioanalyzer), and synthesis of fluorescently tagged cRNA target samples (TotalPrep RNA Amplification Kit; Illumina Inc., San Diego CA). Target cRNA samples were hybridized to Illumina HT-12 v4 BeadArrays by the UCLA Neuroscience Genomics Core Laboratory following the manufacturer’s standard protocol. All RNA isolation and gene expression assay procedures were performed after the total sample set was obtained, and all assays were conducted in a single pass by laboratory personnel blind to participant characteristics and study hypotheses. One sample yielded insufficient cRNA for valid analysis and was deleted in production of the analyzed gene expression data set based on a standard quality assurance test rejecting any sample with a transcriptome-wide median raw expression value < 100. Gene expression data were quantile normalized and log_2_-transformed for statistical analysis. Random variations in Illumina BeadArray production resulted in missing *IL6* determinations for some samples [4], so we conservatively deleted all *IL6* data (rather than attempting to impute missing values), resulting in a 52-gene CTRA score in primary analyses of the confirmation study. (Similar results emerged in analyses using imputed *IL6* data; S6 Table). Data are posted as Gene Expression Omnibus GSE55762.

**Statistical methods**

Associations among continuous variables were assessed by Pearson correlation, associations between continuous and binary variables were assessed by point-biserial correlation, associations between continuous and polytomous variables were quantified by one-way analysis of variance (ANOVA), and associations between two categorical variables were analyzed by *X*^2^ tests. All reported *p*-values represent 2-tailed tests with statistical significance defined by *p* < .05. Potential multicollinearity among variables was quantified by variance inflation factor (VIF) for each regressor: VIF = (1-*R*^2^)^-1^, where *R*^2^ is the coefficient of determination from a multiple regression of each regressor variable on all others [5, 6]. VIF values were computed using SAS (v9.3) PROC REG (SAS Institute, Cary NC) and compared to the established threshold value of 10 to identify material multicollinearity [5, 6].

**Confirmatory factor analysis**

Confirmatory factor analysis (CFA) was employed to quantify the magnitude of reliable unique variance in each MHC-SF scale by comparing the fit of models hypothesizing: (1.) a single common well-being factor, (2.) a 2-d factor structure involving hedonic and eudaimonic domains (i.e., pooling psychological and social well-being items), and (3.) a 3-d factor structure involving dimensions of hedonic, psychological, and social well-being [3, 7-12]. CFA models were fit by maximum likelihood estimation using SAS PROC CALIS. For consistency with gene expression results, we analyzed data from the 122 cases with valid gene expression data and no missing gene expression covariate values. One additional case was deleted due to missing values on 1 of the 14 MHC-SF items, leaving a final analyzed sample size of n = 121. The MHC-SF factor structure has been extensively validated in previous research involving large and diverse samples [7-12], and the MHC-SF was developed to maximize theoretical domain coverage rather than psychometric cohesion [3, 13]. As such, we made no attempt to re-engineer the MHC-SF scales by altering item-factor allocations based on the data at hand. (Ad hoc item reallocation is prone to capitalize on chance and yield unreliable findings due to the greater sampling variability in item loading parameter estimates compared to overall model fit statistics.[14, 15]) CFA was conducted solely to verify that MHC-SF scales showed significant reliability in the present data set (a significant ratio of measurement consistency to the sample-specific variance in true scores) and to evaluate the relative fit of alternative factor structures involving 1, 2, or 3 a priori-defined scales [3]. Based on published guidelines [14], this data set is sufficiently large to support CFA analysis of 14 items loading on ≤ 3 a priori-defined factors as specified in the MHC-SF scoring guidelines [3]. S1 Table shows results of CFA analyses, and SAS code implementing these analyses is included below in Appendix 1.

**CTRA gene expression analysis**

Gene expression data were analyzed using mixed effect linear models [16] treating the 52 CTRA indicator transcripts as repeated measurements tested for common average association with well-being scores. All associations were adjusted for potential confounding by the same set of a priori-selected covariates as in [1]: age, sex, white vs. non-white race, BMI, alcohol consumption, smoking, current illness symptoms, and 8 RNA transcripts indicating the relative prevalence of CD4+ and CD8+ T lymphocytes (*CD3D*, *CD3E*, *CD4*, *CD8A*), B lymphocytes (*CD19*), NK cells (*FCGR3A*, *NCAM1*), and monocytes (*CD14*) in the assayed PBMC pools. Analyses also included a fixed effect of gene to control for transcript-specific heterogeneity (genes were not randomly sampled or exchangeable). Covariates were selected a priori based on (1.) matching the analytic model in [1], and, (2.) addressing the same substantive rationales noted for covariate selection in the discovery study – previously observed association with PBMC gene expression profiles and/or protein biomarkers of inflammation, interferon response, and antibody production [17-25], and transcript covariates encoding the canonical immunologic marker proteins for each of the major leukocyte subsets [26].

The form of statistical analysis models was specified a priori based on, (1.) matching the set of covariates and well-being measures employed in the discovery study analysis, (2.) addressing new substantive questions regarding distinct associations for psychological and social well-being dimensions (2-d eudaimonia domain within the 3-d representation of well-being), and associations involving 1-d continuous measures of total well-being and categorical definition of overall flourishing mental health [3, 27], and, (3.) application of mixed effect linear modeling to account for correlated residuals across CTRA gene transcripts. The basic analysis model is:

(Eq 1.) Expression = Gene + Male + Age + White + BMI + IllnessSymptoms + AlcoholHistory

+ SmokingHistory + *CD3D* + *CD3E* + *CD4* + *CD8A* + *CD19* + *FCGR3A* + *NCAM1* + *CD14*

+ HedonicWellBeing + EudaimonicWellBeing + Residual ~ (0, **R**),

where **R** denotes the variance-covariance matrix for the 52 CTRA indicator genes and Expression represents standardized values of log_2_-transformed quantile normalized gene expression values. Mixed linear models were estimated using SAS PROC MIXED [28], treating Gene as a fixed repeated measure effect with residual covariance modeled by a heterogeneous compound symmetry covariance matrix. The 53-parameter heterogeneous compound symmetry structure (52 variance parameters + 1 average correlation factor) was selected for the **R** matrix because it efficiently accounted for heteroscedasticity across genes and general residual correlation to facilitate accurate estimation of sampling variability of regression parameters relating average CTRA gene expression levels to fixed effect well-being scores. We also evaluated fully unstructured covariance matrices that attempted to directly estimate each of the 1,326 unique covariance parameters. However, the PROC MIXED Newton-Raphson estimation algorithm often failed to converge to a valid solution for unstructured **R** matrices, and, when it did converge, often yielded unstable or invalid parameter estimates due to the technical demands of estimating the 1,326 individual covariance parameters in addition to the 52 variance parameters (yielding a relatively low 4.6-fold support ratio relative to the total 6,344 gene expression observations available in this data set). Following the manufacturer’s guidance [28, 29], the 53-parameter heterogeneous compound symmetry structure was selected for primary analyses because it allowed for more efficient estimation of heterogeneous covariance among genes while consistently yielding valid parameter estimates (supported by a more robust 119.7-fold support ratio relative to observations). (Like unstructured **R** matrices, heterogeneous compound symmetry **R** matrices adjust the estimated sampling variability of regression coefficients to account for covariance among the 52 CTRA indicator transcripts by estimating their 1,326 unique covariances and 52 variances. However, the heterogeneous compound symmetry specification accomplishes this objective more efficiently by estimating the average correlation among genes and then utilizing the implied set of 1,326 distinct covariance parameters in calculation of standard errors for fixed effect regression coefficients, rather than attempting to directly estimate each of the 1,326 individual covariances in isolation. Fixed effect standard errors and test statistics are relatively robust to such mild **R** matrix underspecification at this sample size [30].) All model parameters were estimated by maximum likelihood because our substantive interest lay in the fixed effects associating gene expression with well-being scores [28, 29]. Ancillary sensitivity analyses also examined the effects of alternative restricted maximum likelihood and minimum variance quadratic unbiased estimation on fixed effect parameter estimates in models specifying unstructured **R** matrices (S4 Table). To facilitate convergence of estimation algorithms, quantile-normalized log_2_-transformed gene expression values were standardized to mean = 0, SD = 1 within gene to reduce substantial heteroscedasticity across genes. Brown-Forsythe homogeneity analyses implemented in SAS PROC GLM showed > 10-fold variation in the SD of basic quantile-normalized, log2-transformed gene expression values (rejecting the homogeneous variance hypothesis at *p* < .0001). Absent standardization, heteroscedasticity induced non-positive definite Hessian matrices and prevented convergence of parameter estimation algorithms [28]. Following standardization within gene, standard score signs were inverted for negative elements of the CTRA gene set (31 interferon-related genes and 3 antibody-related genes) as previously described [1]. Missing behavioral data (n = 44; 26 cases missing MHC-SF responses, an additional 15 missing smoking history, and an additional 3 missing alcohol history) and invalid RNA assay results (n = 1, among those missing demographic and behavioral data) reduced the analyzed gene expression data set for primary analyses from a total of 166 blood samples to n = 122 cases assessed on 52 CTRA transcripts, for a total of 6,344 analyzed gene expression values. This yields support ratios of 119.7 observations per covariance parameter and 89.4-91.9 observations per fixed effect parameter depending on the specific well-being representation analyzed (i.e., analyses substituting for the 2-d well-being representation above a 1-d continuous measure of total well-being, a binary categorical representation of flourishing mental health, or a 3-d representation of hedonic, psychological, and social well-being). SAS code implementing primary analyses is included below in Appendix 2.

To facilitate biological interpretation of associations in their natural metric of relative transcript abundance, point estimates of association were also generated for non-standardized log_2_-gene expression values as in [1]. Results were graphed in Fig 1C as regressor range-spanning association coefficients in the % difference metric (i.e., multiplying simple association coefficients specifying the derivative of gene expression with respect to a 1-SD difference in well-being to the more intuitive metric of the derivative with respect to a 4-SD difference in well-being, corresponding to the expected difference in CTRA gene expression for individuals at the low end of the observed range of well-being values [-2 SD relative to mean] vs. those at the high end of the observed range of well-being values [+2 SD relative to mean], followed by base 2 exponentiation and subtraction from the null hypothesis value of 1). A 4-SD range approximates the empirically observed range of well-being scores (Fig 1A-B). Fig. 1C shows results comparing average CTRA associations with hedonic and eudaimonic well-being scores in discovery and confirmation samples.

**Pooled sample analyses**

To provide aggregate estimates of association between CTRA gene expression and MHC-SF well-being based on all data currently available, we also conducted pooled analyses of the combined discovery and confirmation samples. Analyses were performed using the model specified above with an additional covariate adjusting for systematic differences across studies. Ancillary analyses included Study x Well-being interaction terms to determine whether average association coefficients differed significantly across study samples. Primary pooled sample analyses included *IL6* data available in the discovery study, and similar results emerged in analyses deleting all *IL6* values from the pooled data set. These analyses involved a total n = 198 cases assessed on either 53 (discovery) or 52 (confirmation) CTRA gene transcripts for a total of 10,372 gene expression observations, yielding a support ratio of 195.7 observations per **R** matrix parameter and > 140 observations per fixed effect parameter across alternative representations of well-being.

**References**

**Appendix 1 – SAS code for CFA analyses.**

data mhcsf_items;

infile 'SOBC2_MHCSF_items.txt';

input PID $ CHAR10. SF1 SF2 SF3 SF4 SF5 SF6 SF7 SF8 SF9 SF10 SF11 SF12 SF13 SF14;

proc means n nmiss mean stddev min max;

var SF1-SF14;

proc print uniform;

var PID SF1-SF14;

proc corr alpha nomiss;

title 'Reliability of Hedonic well-being scores';

var sf1-sf3;

proc corr alpha nomiss;

title 'Reliability of Eudaimonic well-being scores';

var sf4-sf14;

proc corr alpha nomiss;

title 'Reliaby of Social well-being scores';

var sf4-sf8;

proc corr alpha nomiss;

title 'Reliability of Psychological well-being scores';

var sf9-sf14;

proc corr alpha nomiss;

title 'Reliability of TotalMHC well-being scores';

var sf1-sf14;

proc corr alpha nomiss;

title 'Reliability of Brown alternative Psychological well-being scores';

var sf4-sf5 sf9-sf14;

proc corr alpha nomiss;

title 'Reliability of Brown alternative Social well-being scores';

var sf6-sf8;

* Generating non-missing data matrix for CFA. ;

proc corr outp=sfcorr2 noprint nomiss;

var sf1-sf14;

* Confirmatory factor analyses. ;

proc calis data=sfcorr2;

title 'CFA - 1 factor model – General well-being';

factor

wellbeing > sf1-sf14;

pvar

wellbeing = 1.;

proc calis data=sfcorr2;

title 'CFA - 2 factor model - Hedonic + Eudaimonic';

factor

hed > sf1-sf3,

eud > sf4-sf14;

pvar

hed eud = 2 * 1.;

proc calis data=sfcorr2;

title 'CFA - 3 factor model - Hedonic + Psychological + Social';

factor

hed > sf1-sf3,

swb > sf4-sf8,

pwb > sf9-sf14;

pvar

hed swb pwb = 3 * 1.;

proc calis data=sfcorr2;

title 'CFA - Brown 2 factor model – Brown general well-being + Brown social';

factor

BrownSWB > sf6-sf8,

WB > sf1-sf5 sf9-sf14;

pvar

BrownSWB WB = 2 * 1.;

proc calis data=sfcorr2;

title 'CFA - Brown 3 factor model - Hedonic + Brown social + Brown psychological';

factor

hed > sf1-sf3,

BrownSWB > sf6-sf8,

BrownPWB > sf4-sf5 sf9-sf14;

pvar

hed BrownSWB BrownPWB = 3 * 1.;

**Appendix 2 – SAS code for CTRA gene expression analyses.**

* Reading Study 1 regressors from space-delimited text file with header removed and PID trimmed to 10 characters. ;

data regressors_study1;

infile 'GSE45330_Regressors.txt';

input PID $ CHAR10. ZHedonic ZEudaimonic Hedonic Eudaimonic SWB PWB

TotalMHC Flourish BrownPWB BrownSWB Male Age White BMI Alcohol Smoke

Ill CD3D CD3E CD4 CD8A CD19 FCGR3A NCAM1 CD14;

Study = 1;

proc sort;

by PID;

* Reading Study 2 regressors from space-delimited text file with header removed and PID trimmed to 10 characters. ;

data regressors_study2;

infile 'GSE55762_Regressors.txt';

input PID $ CHAR10. Hedonic Eudaimonic SWB PWB TotalMHC Flourish

BrownPWB BrownSWB Age White Male BMI Ill Smoke Alcohol CESD CD3E CD3D CD19

CD4 CD8A FCGR3A NCAM1 CD14;

Study = 2;

proc sort;

by PID;

* Forming pooled regressor matrix. ;

data regressors;

set regressors_study1 regressors_study2;

* Deleting cases with missing covariate data. ;

if Male = . then delete;

if Age = . then delete;

if White = . then delete;

if BMI = . then delete;

if Smoke = . then delete;

if Alcohol = . then delete;

if Ill = . then delete;

* Standardizing continuous well-being scores. ;

proc standard m=0 s=1 data=regressors out=regressors_z;

var Hedonic Eudaimonic PWB SWB TotalMHC BrownPWB BrownSWB;

* Reading Study 1 gene expression data from space-delimited text file. ;

data genes_study1;

infile '~/consult/fredrickson/pooled/final/GSE45330_NormalizedExpression.txt';

input Gene $ SOBC1_1283 SOBC1_1252 SOBC1_1235 SOBC1_1285 SOBC1_1255

SOBC1_1601 SOBC1_1199 SOBC1_1480 SOBC1_1259 SOBC1_1256 SOBC1_1271

SOBC1_1220 SOBC1_1225 SOBC1_1194 SOBC1_1333 SOBC1_1192 SOBC1_1260

SOBC1_1188 SOBC1_1196 SOBC1_1186 SOBC1_1326 SOBC1_1277 SOBC1_1231

SOBC1_1195 SOBC1_1197 SOBC1_1300 SOBC1_1232 SOBC1_1441 SOBC1_1239

SOBC1_1264 SOBC1_1185 SOBC1_1262 SOBC1_1306 SOBC1_1345 SOBC1_1343

SOBC1_1297 SOBC1_1329 SOBC1_1245 SOBC1_1293 SOBC1_1189 SOBC1_1241

SOBC1_1292 SOBC1_1328 SOBC1_1228 SOBC1_1294 SOBC1_1288 SOBC1_1580

SOBC1_1422 SOBC1_1681 SOBC1_1344 SOBC1_1273 SOBC1_1223 SOBC1_1720

SOBC1_1721 SOBC1_1287 SOBC1_1237 SOBC1_1440 SOBC1_1286 SOBC1_1740

SOBC1_1760 SOBC1_1900 SOBC1_1247 SOBC1_1190 SOBC1_1201 SOBC1_1305

SOBC1_1193 SOBC1_1246 SOBC1_1182 SOBC1_1263 SOBC1_1234 SOBC1_1198

SOBC1_1299 SOBC1_1680 SOBC1_1600 SOBC1_1722 SOBC1_1683 SOBC1_1684

SOBC1_1289 SOBC1_1880;

* Removing data for genes not used. ;

keep = 0;

if gene = 'FOS' then keep = 1;

if gene = 'FOSB' then keep = 1;

if gene = 'FOSL1' then keep = 1;

if gene = 'FOSL2' then keep = 1;

if gene = 'GBP1' then keep = 1;

if gene = 'IFI16' then keep = 1;

if gene = 'IFI27' then keep = 1;

if gene = 'IFI27L1' then keep = 1;

if gene = 'IFI27L2' then keep = 1;

if gene = 'IFI30' then keep = 1;

if gene = 'IFI35' then keep = 1;

if gene = 'IFI44' then keep = 1;

if gene = 'IFI44L' then keep = 1;

if gene = 'IFI6' then keep = 1;

if gene = 'IFIH1' then keep = 1;

if gene = 'IFIT1' then keep = 1;

if gene = 'IFIT1L' then keep = 1;

if gene = 'IFIT2' then keep = 1;

if gene = 'IFIT3' then keep = 1;

if gene = 'IFIT5' then keep = 1;

if gene = 'IFITM1' then keep = 1;

if gene = 'IFITM2' then keep = 1;

if gene = 'IFITM3' then keep = 1;

if gene = 'IFITM4P' then keep = 1;

if gene = 'IFITM5' then keep = 1;

if gene = 'IFNB1' then keep = 1;

if gene = 'IGJ' then keep = 1;

if gene = 'IGLL1' then keep = 1;

if gene = 'IGLL3' then keep = 1;

if gene = 'IL1A' then keep = 1;

if gene = 'IL1B' then keep = 1;

if gene = 'IL6' then keep = 1;

if gene = 'IL8' then keep = 1;

if gene = 'IRF2' then keep = 1;

if gene = 'IRF7' then keep = 1;

if gene = 'IRF8' then keep = 1;

if gene = 'JUN' then keep = 1;

if gene = 'JUNB' then keep = 1;

if gene = 'JUND' then keep = 1;

if gene = 'MX1' then keep = 1;

if gene = 'MX2' then keep = 1;

if gene = 'NFKB1' then keep = 1;

if gene = 'NFKB2' then keep = 1;

if gene = 'OAS1' then keep = 1;

if gene = 'OAS2' then keep = 1;

if gene = 'OAS3' then keep = 1;

if gene = 'OASL' then keep = 1;

if gene = 'PTGS1' then keep = 1;

if gene = 'PTGS2' then keep = 1;

if gene = 'REL' then keep = 1;

if gene = 'RELA' then keep = 1;

if gene = 'RELB' then keep = 1;

if gene = 'TNF' then keep = 1;

if keep ne 1 then delete;

proc sort;

by gene;

* Reading Study 2 gene expression data from space-delimited text file. ;

data genes_study2;

infile '~/consult/fredrickson/pooled/final/GSE55762_NormalizedExpression.txt';

input Gene $ SOBC2_2112 SOBC2_2037 SOBC2_2072 SOBC2_2147 SOBC2_2076

SOBC2_2093 SOBC2_2020 SOBC2_2121 SOBC2_2048 SOBC2_2073 SOBC2_2023

SOBC2_2086 SOBC2_2202 SOBC2_2091 SOBC2_2104 SOBC2_2074 SOBC2_2044

SOBC2_2089 SOBC2_2028 SOBC2_2144 SOBC2_2180 SOBC2_2123 SOBC2_2036

SOBC2_2113 SOBC2_2187 SOBC2_2047 SOBC2_2168 SOBC2_2096 SOBC2_2042

SOBC2_2166 SOBC2_2021 SOBC2_2031 SOBC2_2030 SOBC2_2167 SOBC2_2039

SOBC2_2032 SOBC2_2119 SOBC2_2049 SOBC2_2171 SOBC2_2169 SOBC2_2026

SOBC2_2131 SOBC2_2145 SOBC2_2024 SOBC2_2106 SOBC2_2201 SOBC2_2183

SOBC2_2064 SOBC2_2092 SOBC2_2035 SOBC2_2043 SOBC2_2182 SOBC2_2061

SOBC2_2079 SOBC2_2025 SOBC2_2136 SOBC2_2066 SOBC2_2639 SOBC2_2597

SOBC2_2593 SOBC2_2603 SOBC2_2566 SOBC2_2637 SOBC2_2586 SOBC2_2592

SOBC2_2629 SOBC2_2569 SOBC2_2567 SOBC2_2625 SOBC2_2607 SOBC2_2613

SOBC2_2574 SOBC2_2605 SOBC2_2663 SOBC2_2647 SOBC2_2564 SOBC2_2599

SOBC2_2575 SOBC2_2661 SOBC2_2675 SOBC2_2560 SOBC2_2638 SOBC2_2636

SOBC2_2654 SOBC2_2579 SOBC2_2619 SOBC2_2659 SOBC2_2610 SOBC2_2623

SOBC2_2606 SOBC2_2601 SOBC2_2626 SOBC2_2618 SOBC2_2561 SOBC2_2644

SOBC2_2635 SOBC2_2550 SOBC2_2602 SOBC2_2554 SOBC2_2616 SOBC2_2648

SOBC2_2594 SOBC2_2630 SOBC2_2622 SOBC2_2736 SOBC2_2609 SOBC2_2627

SOBC2_2728 SOBC2_2632 SOBC2_2587 SOBC2_2596 SOBC2_2723 SOBC2_2714

SOBC2_2642 SOBC2_2176 SOBC2_2186 SOBC2_2108 SOBC2_2165 SOBC2_2174

SOBC2_2205 SOBC2_2097 SOBC2_2094 ;

* Removing data for genes not used. ;

keep = 0;

if gene = 'FOS' then keep = 1;

if gene = 'FOSB' then keep = 1;

if gene = 'FOSL1' then keep = 1;

if gene = 'FOSL2' then keep = 1;

if gene = 'GBP1' then keep = 1;

if gene = 'IFI16' then keep = 1;

if gene = 'IFI27' then keep = 1;

if gene = 'IFI27L1' then keep = 1;

if gene = 'IFI27L2' then keep = 1;

if gene = 'IFI30' then keep = 1;

if gene = 'IFI35' then keep = 1;

if gene = 'IFI44' then keep = 1;

if gene = 'IFI44L' then keep = 1;

if gene = 'IFI6' then keep = 1;

if gene = 'IFIH1' then keep = 1;

if gene = 'IFIT1' then keep = 1;

if gene = 'IFIT1L' then keep = 1;

if gene = 'IFIT2' then keep = 1;

if gene = 'IFIT3' then keep = 1;

if gene = 'IFIT5' then keep = 1;

if gene = 'IFITM1' then keep = 1;

if gene = 'IFITM2' then keep = 1;

if gene = 'IFITM3' then keep = 1;

if gene = 'IFITM4P' then keep = 1;

if gene = 'IFITM5' then keep = 1;

if gene = 'IFNB1' then keep = 1;

if gene = 'IGJ' then keep = 1;

if gene = 'IGLL1' then keep = 1;

if gene = 'IGLL3' then keep = 1;

if gene = 'IL1A' then keep = 1;

if gene = 'IL1B' then keep = 1;

if gene = 'IL6' then keep = 1;

if gene = 'IL8' then keep = 1;

if gene = 'IRF2' then keep = 1;

if gene = 'IRF7' then keep = 1;

if gene = 'IRF8' then keep = 1;

if gene = 'JUN' then keep = 1;

if gene = 'JUNB' then keep = 1;

if gene = 'JUND' then keep = 1;

if gene = 'MX1' then keep = 1;

if gene = 'MX2' then keep = 1;

if gene = 'NFKB1' then keep = 1;

if gene = 'NFKB2' then keep = 1;

if gene = 'OAS1' then keep = 1;

if gene = 'OAS2' then keep = 1;

if gene = 'OAS3' then keep = 1;

if gene = 'OASL' then keep = 1;

if gene = 'PTGS1' then keep = 1;

if gene = 'PTGS2' then keep = 1;

if gene = 'REL' then keep = 1;

if gene = 'RELA' then keep = 1;

if gene = 'RELB' then keep = 1;

if gene = 'TNF' then keep = 1;

if keep ne 1 then delete;

proc sort;

by gene;

* Forming pooled gene expression matrix. ;

data genes;

merge genes_study1 genes_study2;

by gene;

* Transposing gene expression matrix to PID x Gene format. ;

proc transpose data=genes name = PID out=genes_horiz;

id Gene;

var SOBC1_1283 SOBC1_1252 SOBC1_1235 SOBC1_1285 SOBC1_1255 SOBC1_1601

SOBC1_1199 SOBC1_1480 SOBC1_1259 SOBC1_1256 SOBC1_1271 SOBC1_1220

SOBC1_1225 SOBC1_1194 SOBC1_1333 SOBC1_1192 SOBC1_1260 SOBC1_1188

SOBC1_1196 SOBC1_1186 SOBC1_1326 SOBC1_1277 SOBC1_1231 SOBC1_1195

SOBC1_1197 SOBC1_1300 SOBC1_1232 SOBC1_1441 SOBC1_1239 SOBC1_1264

SOBC1_1185 SOBC1_1262 SOBC1_1306 SOBC1_1345 SOBC1_1343 SOBC1_1297

SOBC1_1329 SOBC1_1245 SOBC1_1293 SOBC1_1189 SOBC1_1241 SOBC1_1292

SOBC1_1328 SOBC1_1228 SOBC1_1294 SOBC1_1288 SOBC1_1580 SOBC1_1422

SOBC1_1681 SOBC1_1344 SOBC1_1273 SOBC1_1223 SOBC1_1720 SOBC1_1721

SOBC1_1287 SOBC1_1237 SOBC1_1440 SOBC1_1286 SOBC1_1740 SOBC1_1760

SOBC1_1900 SOBC1_1247 SOBC1_1190 SOBC1_1201 SOBC1_1305 SOBC1_1193

SOBC1_1246 SOBC1_1182 SOBC1_1263 SOBC1_1234 SOBC1_1198 SOBC1_1299

SOBC1_1680 SOBC1_1600 SOBC1_1722 SOBC1_1683 SOBC1_1684 SOBC1_1289

SOBC1_1880 SOBC2_2112 SOBC2_2037 SOBC2_2072 SOBC2_2147 SOBC2_2076

SOBC2_2093 SOBC2_2020 SOBC2_2121 SOBC2_2048 SOBC2_2073 SOBC2_2023

SOBC2_2086 SOBC2_2202 SOBC2_2091 SOBC2_2104 SOBC2_2074 SOBC2_2044

SOBC2_2089 SOBC2_2028 SOBC2_2144 SOBC2_2180 SOBC2_2123 SOBC2_2036

SOBC2_2113 SOBC2_2187 SOBC2_2047 SOBC2_2168 SOBC2_2096 SOBC2_2042

SOBC2_2166 SOBC2_2021 SOBC2_2031 SOBC2_2030 SOBC2_2167 SOBC2_2039

SOBC2_2032 SOBC2_2119 SOBC2_2049 SOBC2_2171 SOBC2_2169 SOBC2_2026

SOBC2_2131 SOBC2_2145 SOBC2_2024 SOBC2_2106 SOBC2_2201 SOBC2_2183

SOBC2_2064 SOBC2_2092 SOBC2_2035 SOBC2_2043 SOBC2_2182 SOBC2_2061

SOBC2_2079 SOBC2_2025 SOBC2_2136 SOBC2_2066 SOBC2_2639 SOBC2_2597

SOBC2_2593 SOBC2_2603 SOBC2_2566 SOBC2_2637 SOBC2_2586 SOBC2_2592

SOBC2_2629 SOBC2_2569 SOBC2_2567 SOBC2_2625 SOBC2_2607 SOBC2_2613

SOBC2_2574 SOBC2_2605 SOBC2_2663 SOBC2_2647 SOBC2_2564 SOBC2_2599

SOBC2_2575 SOBC2_2661 SOBC2_2675 SOBC2_2560 SOBC2_2638 SOBC2_2636

SOBC2_2654 SOBC2_2579 SOBC2_2619 SOBC2_2659 SOBC2_2610 SOBC2_2623

SOBC2_2606 SOBC2_2601 SOBC2_2626 SOBC2_2618 SOBC2_2561 SOBC2_2644

SOBC2_2635 SOBC2_2550 SOBC2_2602 SOBC2_2554 SOBC2_2616 SOBC2_2648

SOBC2_2594 SOBC2_2630 SOBC2_2622 SOBC2_2736 SOBC2_2609 SOBC2_2627

SOBC2_2728 SOBC2_2632 SOBC2_2587 SOBC2_2596 SOBC2_2723 SOBC2_2714

SOBC2_2642 SOBC2_2176 SOBC2_2186 SOBC2_2108 SOBC2_2165 SOBC2_2174

SOBC2_2205 SOBC2_2097 SOBC2_2094 ;

proc sort;

by PID;

* Merging Gene expression matrix with Regressor matrix. ;

data horiz;

merge regressors_z genes_horiz;

by PID;

* Removing gene expression cases with no covariate data available. ;

if Study = . then delete;

proc sort;

by PID Study Male Age White BMI Alcohol Smoke Ill CD3D CD3E CD4 CD8A

CD19 FCGR3A NCAM1 CD14 Hedonic Eudaimonic PWB SWB Flourish TotalMHC

BrownPWB BrownSWB CESD;

* Transposing to stacked-Gene format for PROC MIXED. ;

proc transpose;

var il1a il1b il6 il8 tnf ptgs1 ptgs2 fos fosb fosl1 fosl2 jun junb

jund nfkb1 nfkb2 rel rela relb igj igll1 igll3 gbp1 ifi16 ifi27

ifi27l1 ifi27l2 ifi30 ifi35 ifi44 ifi44l ifi6 ifih1 ifit1 ifit1l ifit2

ifit3 ifit5 ifitm1 ifitm2 ifitm3 ifitm4p ifitm5 ifnb1 irf8 irf2 irf7

mx1 mx2 oas1 oas2 oas3 oasl;

by PID Study Male Age White BMI Alcohol Smoke Ill CD3D CD3E CD4 CD8A

CD19 FCGR3A NCAM1 CD14 Hedonic Eudaimonic PWB SWB Flourish TotalMHC

BrownPWB BrownSWB CESD;

data vertical;

set;

Gene = _NAME_;

* Log2-transform gene expression values. ;

Expression = log2(COL1);

proc sort data=vertical;

by Gene Study;

* Standardize gene expression values to mitigate heteroscedasticity. ;

proc standard m=0 s=1 data=vertical out=vertical_z;

var Expression;

by Gene Study;

* Reverse sign of standardized expression values for negative elements of CTRA: Type I Interferon and Antibody genes. ;

data pooled_z;

set vertical_z;

if Gene ne 'IL1A'

and Gene ne 'IL1B'

and Gene ne 'IL6'

and Gene ne 'IL8'

and Gene ne 'TNF'

and Gene ne 'PTGS1'

and Gene ne 'PTGS2'

and Gene ne 'FOS'

and Gene ne 'FOSB'

and Gene ne 'FOSL1'

and Gene ne 'FOSL2'

and Gene ne 'JUN'

and Gene ne 'JUNB'

and Gene ne 'JUND'

and Gene ne 'NFKB1'

and Gene ne 'NFKB2'

and Gene ne 'REL'

and Gene ne 'RELA'

and Gene ne 'RELB'

then Expression = -1*Expression;

proc sort;

by PID Gene;

* Assemble data set for Study 2 only. ;

data study2;

set pooled_z;

* Removing data from Study 1. ;

if Study = 1 then delete;

* Removing missing IL6 data = all IL6 data in Study 2. ;

if Gene = 'IL6' and Expression = . then delete;

* Re-standardizing continuous well-being scores for Study 2 alone. ;

proc standard m=0 s=1 data=study2 out=study2_z;

var Hedonic Eudaimonic PWB SWB TotalMHC BrownPWB BrownSWB;

data study2_regressors;

set study2_z;

* Remove redundant Gene-specific regressor submatrices within stacked data matrix. ;

if Gene ne 'IL1B' then delete;

* Characterizing Study 2-specific regressor matrix. ;

proc means data=study2_regressors n nmiss mean stddev min max;

title 'Study 2: Descriptive statistics for final vertical data matrix';

var Male Age White BMI Alcohol Smoke Ill CD3D CD3E CD4 CD8A CD19

FCGR3A NCAM1 CD14 Hedonic Eudaimonic PWB SWB Flourish TotalMHC

BrownSWB BrownPWB;

proc chart data=study2_regressors;

hbar Male White Alcohol Smoke Flourish / discrete;

proc univariate data=study2_regressors plot;

var Hedonic Eudaimonic PWB SWB TotalMHC BrownSWB BrownPWB;

proc print data=study2_regressors uniform;

title 'Study 2: Final regressor matrix';

var Study PID Male Age White BMI Alcohol Smoke Ill Hedonic Eudaimonic

Flourish TotalMHC PWB SWB BrownSWB BrownPWB CD3D CD3E CD4 CD8A CD19 FCGR3A NCAM1 CD14;

proc corr data=study2_regressors pearson fisher;

title 'Study 2: Regressor matrix characterization - simple correlations';

var Hedonic Eudaimonic PWB SWB TotalMHC BrownSWB BrownPWB;

proc corr data=study2_regressors pearson fisher;

title 'Study 2: Potential confounder characterization';

var Hedonic Eudaimonic PWB SWB Flourish TotalMHC BrownSWB BrownPWB;

with Male Age White BMI Alcohol Smoke Ill CD3D CD3E CD4 CD8A CD19 FCGR3A NCAM1 CD14;

proc reg data=study2_regressors;

title 'Study 2: Regressor matrix characterization - Hedonic + Eudaimonic';

model Expression = Male Age White BMI Alcohol Smoke Ill CD3D CD3E CD4 CD8A

CD19 FCGR3A NCAM1 CD14 Hedonic Eudaimonic / tol vif;

proc reg data=study2_regressors;

title 'Study 2: Regressor matrix characterization - Hedonic + PWB + SWB';

model Expression = Male Age White BMI Alcohol Smoke Ill CD3D CD3E CD4 CD8A

CD19 FCGR3A NCAM1 CD14 Hedonic PWB SWB / tol vif;

proc reg data=study2_regressors;

title 'Study 2: Regressor matrix characterization - Flourish';

model Expression = Male Age White BMI Alcohol Smoke Ill CD3D CD3E CD4 CD8A

CD19 FCGR3A NCAM1 CD14 Flourish / tol vif;

proc reg data=study2_regressors;

title 'Study 2: Regressor matrix characterization - TotalMHC';

model Expression = Male Age White BMI Alcohol Smoke Ill CD3D CD3E CD4 CD8A

CD19 FCGR3A NCAM1 CD14 TotalMHC / tol vif;

proc reg data=study2_regressors;

title 'Regressor matrix characterization - Hedonic + BrownPWB + BrownSWB';

model Expression = Male Age White BMI Alcohol Smoke Ill CD3D CD3E CD4 CD8A

CD19 FCGR3A NCAM1 CD14 Hedonic BrownPWB BrownSWB / tol vif;

* Mixed linear model analyses. ;

proc mixed data=study2_z method=ml;

title 'Study 2 - Model 1: Eudaimonic well-being + Hedonic well-being';

class Gene PID;

model Expression = Gene Male Age White BMI Ill Alcohol Smoke

CD3D CD3E CD4 CD8A CD19 FCGR3A NCAM1 CD14 Hedonic Eudaimonic / solution cl;

repeated Gene / subject=PID type=csh;

contrast 'LeukSubsets' CD3D 1 CD3E 0 CD4 0 CD8A 0 CD19 0 FCGR3A 0 NCAM1 0 CD14 0,

CD3D 0 CD3E 1 CD4 0 CD8A 0 CD19 0 FCGR3A 0 NCAM1 0 CD14 0,

CD3D 0 CD3E 0 CD4 1 CD8A 0 CD19 0 FCGR3A 0 NCAM1 0 CD14 0,

CD3D 0 CD3E 0 CD4 0 CD8A 1 CD19 0 FCGR3A 0 NCAM1 0 CD14 0,

CD3D 0 CD3E 0 CD4 0 CD8A 0 CD19 1 FCGR3A 0 NCAM1 0 CD14 0,

CD3D 0 CD3E 0 CD4 0 CD8A 0 CD19 0 FCGR3A 1 NCAM1 0 CD14 0,

CD3D 0 CD3E 0 CD4 0 CD8A 0 CD19 0 FCGR3A 0 NCAM1 1 CD14 0,

CD3D 0 CD3E 0 CD4 0 CD8A 0 CD19 0 FCGR3A 0 NCAM1 0 CD14 1;

contrast 'Well-being' Hedonic 0 Eudaimonic 1,

Hedonic 1 Eudaimonic 0;

estimate 'Eud-Hed' Eudaimonic 1 Hedonic -1;

proc mixed data=study2_z method=ml;

title 'Study 2 - Model 2: Hedonic well-being + Social well-being + Psychological well-being';

class Gene PID;

model Expression = Gene Male Age White BMI Ill Alcohol Smoke

CD3D CD3E CD4 CD8A CD19 FCGR3A NCAM1 CD14 Hedonic SWB PWB / solution cl;

repeated Gene / subject=PID type=csh;

contrast 'LeukSubsets' CD3D 1 CD3E 0 CD4 0 CD8A 0 CD19 0 FCGR3A 0 NCAM1 0 CD14 0,

CD3D 0 CD3E 1 CD4 0 CD8A 0 CD19 0 FCGR3A 0 NCAM1 0 CD14 0,

CD3D 0 CD3E 0 CD4 1 CD8A 0 CD19 0 FCGR3A 0 NCAM1 0 CD14 0,

CD3D 0 CD3E 0 CD4 0 CD8A 1 CD19 0 FCGR3A 0 NCAM1 0 CD14 0,

CD3D 0 CD3E 0 CD4 0 CD8A 0 CD19 1 FCGR3A 0 NCAM1 0 CD14 0,

CD3D 0 CD3E 0 CD4 0 CD8A 0 CD19 0 FCGR3A 1 NCAM1 0 CD14 0,

CD3D 0 CD3E 0 CD4 0 CD8A 0 CD19 0 FCGR3A 0 NCAM1 1 CD14 0,

CD3D 0 CD3E 0 CD4 0 CD8A 0 CD19 0 FCGR3A 0 NCAM1 0 CD14 1;

contrast 'Well-being' Hedonic 1 SWB 0 PWB 0,

Hedonic 0 SWB 1 PWB 0,

Hedonic 0 SWB 0 PWB 1;

contrast 'Eudaimonic' SWB 1 PWB 0,

SWB 0 PWB 1;

estimate 'SWB-PWB' SWB 1 PWB -1;

estimate 'SWB-Hedonic' Hedonic -1 SWB 1;

estimate 'PWB-Hedonic' Hedonic -1 PWB 1;

proc mixed data=study2_z method=ml;

title 'Study 2 - Model 3: Social well-being alone';

class Gene PID;

model Expression = Gene Male Age White BMI Ill Alcohol Smoke

CD3D CD3E CD4 CD8A CD19 FCGR3A NCAM1 CD14 SWB / solution cl;

repeated Gene / subject=PID type=csh;

contrast 'LeukSubsets' CD3D 1 CD3E 0 CD4 0 CD8A 0 CD19 0 FCGR3A 0 NCAM1 0 CD14 0,

CD3D 0 CD3E 1 CD4 0 CD8A 0 CD19 0 FCGR3A 0 NCAM1 0 CD14 0,

CD3D 0 CD3E 0 CD4 1 CD8A 0 CD19 0 FCGR3A 0 NCAM1 0 CD14 0,

CD3D 0 CD3E 0 CD4 0 CD8A 1 CD19 0 FCGR3A 0 NCAM1 0 CD14 0,

CD3D 0 CD3E 0 CD4 0 CD8A 0 CD19 1 FCGR3A 0 NCAM1 0 CD14 0,

CD3D 0 CD3E 0 CD4 0 CD8A 0 CD19 0 FCGR3A 1 NCAM1 0 CD14 0,

CD3D 0 CD3E 0 CD4 0 CD8A 0 CD19 0 FCGR3A 0 NCAM1 1 CD14 0,

CD3D 0 CD3E 0 CD4 0 CD8A 0 CD19 0 FCGR3A 0 NCAM1 0 CD14 1;

proc mixed data=study2_z method=ml;

title 'Study 2 - Model 4: Psychological well-being alone';

class Gene PID;

model Expression = Gene Male Age White BMI Ill Alcohol Smoke

CD3D CD3E CD4 CD8A CD19 FCGR3A NCAM1 CD14 PWB / solution cl;

repeated Gene / subject=PID type=csh;

contrast 'LeukSubsets' CD3D 1 CD3E 0 CD4 0 CD8A 0 CD19 0 FCGR3A 0 NCAM1 0 CD14 0,

CD3D 0 CD3E 1 CD4 0 CD8A 0 CD19 0 FCGR3A 0 NCAM1 0 CD14 0,

CD3D 0 CD3E 0 CD4 1 CD8A 0 CD19 0 FCGR3A 0 NCAM1 0 CD14 0,

CD3D 0 CD3E 0 CD4 0 CD8A 1 CD19 0 FCGR3A 0 NCAM1 0 CD14 0,

CD3D 0 CD3E 0 CD4 0 CD8A 0 CD19 1 FCGR3A 0 NCAM1 0 CD14 0,

CD3D 0 CD3E 0 CD4 0 CD8A 0 CD19 0 FCGR3A 1 NCAM1 0 CD14 0,

CD3D 0 CD3E 0 CD4 0 CD8A 0 CD19 0 FCGR3A 0 NCAM1 1 CD14 0,

CD3D 0 CD3E 0 CD4 0 CD8A 0 CD19 0 FCGR3A 0 NCAM1 0 CD14 1;

proc mixed data=study2_z method=ml;

title 'Study 2 - Model 5: Categorical Flourishing';

class Gene PID;

model Expression = Gene Male Age White BMI Ill Alcohol Smoke

CD3D CD3E CD4 CD8A CD19 FCGR3A NCAM1 CD14 Flourish / solution cl;

repeated Gene / subject=PID type=csh;

contrast 'LeukSubsets' CD3D 1 CD3E 0 CD4 0 CD8A 0 CD19 0 FCGR3A 0 NCAM1 0 CD14 0,

CD3D 0 CD3E 1 CD4 0 CD8A 0 CD19 0 FCGR3A 0 NCAM1 0 CD14 0,

CD3D 0 CD3E 0 CD4 1 CD8A 0 CD19 0 FCGR3A 0 NCAM1 0 CD14 0,

CD3D 0 CD3E 0 CD4 0 CD8A 1 CD19 0 FCGR3A 0 NCAM1 0 CD14 0,

CD3D 0 CD3E 0 CD4 0 CD8A 0 CD19 1 FCGR3A 0 NCAM1 0 CD14 0,

CD3D 0 CD3E 0 CD4 0 CD8A 0 CD19 0 FCGR3A 1 NCAM1 0 CD14 0,

CD3D 0 CD3E 0 CD4 0 CD8A 0 CD19 0 FCGR3A 0 NCAM1 1 CD14 0,

CD3D 0 CD3E 0 CD4 0 CD8A 0 CD19 0 FCGR3A 0 NCAM1 0 CD14 1;

proc mixed data=study2_z method=ml;

title 'Study 2 - Model 6: TotalMHC';

class Gene PID;

model Expression = Gene Male Age White BMI Ill Alcohol Smoke

CD3D CD3E CD4 CD8A CD19 FCGR3A NCAM1 CD14 TotalMHC / solution cl;

repeated Gene / subject=PID type=csh;

contrast 'LeukSubsets' CD3D 1 CD3E 0 CD4 0 CD8A 0 CD19 0 FCGR3A 0 NCAM1 0 CD14 0,

CD3D 0 CD3E 1 CD4 0 CD8A 0 CD19 0 FCGR3A 0 NCAM1 0 CD14 0,

CD3D 0 CD3E 0 CD4 1 CD8A 0 CD19 0 FCGR3A 0 NCAM1 0 CD14 0,

CD3D 0 CD3E 0 CD4 0 CD8A 1 CD19 0 FCGR3A 0 NCAM1 0 CD14 0,

CD3D 0 CD3E 0 CD4 0 CD8A 0 CD19 1 FCGR3A 0 NCAM1 0 CD14 0,

CD3D 0 CD3E 0 CD4 0 CD8A 0 CD19 0 FCGR3A 1 NCAM1 0 CD14 0,

CD3D 0 CD3E 0 CD4 0 CD8A 0 CD19 0 FCGR3A 0 NCAM1 1 CD14 0,

CD3D 0 CD3E 0 CD4 0 CD8A 0 CD19 0 FCGR3A 0 NCAM1 0 CD14 1;

proc mixed data=study2_z method=ml;

title 'Study 2 - model 7: Hedonic well-being + BrownPWB + BrownSWB';

class Gene PID;

model Expression = Gene Male Age White BMI Ill Alcohol Smoke

CD3D CD3E CD4 CD8A CD19 FCGR3A NCAM1 CD14 Hedonic BrownPWB BrownSWB / solution cl;

repeated Gene / subject=PID type=csh;

contrast 'LeukSubsets' CD3D 1 CD3E 0 CD4 0 CD8A 0 CD19 0 FCGR3A 0 NCAM1 0 CD14 0,

CD3D 0 CD3E 1 CD4 0 CD8A 0 CD19 0 FCGR3A 0 NCAM1 0 CD14 0,

CD3D 0 CD3E 0 CD4 1 CD8A 0 CD19 0 FCGR3A 0 NCAM1 0 CD14 0,

CD3D 0 CD3E 0 CD4 0 CD8A 1 CD19 0 FCGR3A 0 NCAM1 0 CD14 0,

CD3D 0 CD3E 0 CD4 0 CD8A 0 CD19 1 FCGR3A 0 NCAM1 0 CD14 0,

CD3D 0 CD3E 0 CD4 0 CD8A 0 CD19 0 FCGR3A 1 NCAM1 0 CD14 0,

CD3D 0 CD3E 0 CD4 0 CD8A 0 CD19 0 FCGR3A 0 NCAM1 1 CD14 0,

CD3D 0 CD3E 0 CD4 0 CD8A 0 CD19 0 FCGR3A 0 NCAM1 0 CD14 1;

contrast 'Well-being' Hedonic 1 BrownPWB 0 BrownSWB 0,

Hedonic 0 BrownPWB 1 BrownSWB 0,

Hedonic 0 BrownPWB 0 BrownSWB 1;

contrast 'Eudaimonic' BrownPWB 1 BrownSWB 0,

BrownPWB 0 BrownSWB 1;

estimate 'BrownPWB-BrownSWB' BrownPWB 1 BrownSWB -1;

estimate 'BrownPWB-Hedonic' Hedonic -1 BrownPWB 1;

estimate 'BrownSWB-Hedonic' Hedonic -1 BrownSWB 1;

proc mixed data=study2_z method=ml;

title 'Study 2 - model 8: BrownPWB alone';

class Gene PID;

model Expression = Gene Male Age White BMI Ill Alcohol Smoke

CD3D CD3E CD4 CD8A CD19 FCGR3A NCAM1 CD14 BrownPWB / solution cl;

repeated Gene / subject=PID type=csh;

contrast 'LeukSubsets' CD3D 1 CD3E 0 CD4 0 CD8A 0 CD19 0 FCGR3A 0 NCAM1 0 CD14 0,

CD3D 0 CD3E 1 CD4 0 CD8A 0 CD19 0 FCGR3A 0 NCAM1 0 CD14 0,

CD3D 0 CD3E 0 CD4 1 CD8A 0 CD19 0 FCGR3A 0 NCAM1 0 CD14 0,

CD3D 0 CD3E 0 CD4 0 CD8A 1 CD19 0 FCGR3A 0 NCAM1 0 CD14 0,

CD3D 0 CD3E 0 CD4 0 CD8A 0 CD19 1 FCGR3A 0 NCAM1 0 CD14 0,

CD3D 0 CD3E 0 CD4 0 CD8A 0 CD19 0 FCGR3A 1 NCAM1 0 CD14 0,

CD3D 0 CD3E 0 CD4 0 CD8A 0 CD19 0 FCGR3A 0 NCAM1 1 CD14 0,

CD3D 0 CD3E 0 CD4 0 CD8A 0 CD19 0 FCGR3A 0 NCAM1 0 CD14 1;

proc mixed data=study2_z method=ml;

title 'Study 2 - model 9: BrownSWB alone';

class Gene PID;

model Expression = Gene Male Age White BMI Ill Alcohol Smoke

CD3D CD3E CD4 CD8A CD19 FCGR3A NCAM1 CD14 BrownSWB / solution cl;

repeated Gene / subject=PID type=csh;

contrast 'LeukSubsets' CD3D 1 CD3E 0 CD4 0 CD8A 0 CD19 0 FCGR3A 0 NCAM1 0 CD14 0,

CD3D 0 CD3E 1 CD4 0 CD8A 0 CD19 0 FCGR3A 0 NCAM1 0 CD14 0,

CD3D 0 CD3E 0 CD4 1 CD8A 0 CD19 0 FCGR3A 0 NCAM1 0 CD14 0,

CD3D 0 CD3E 0 CD4 0 CD8A 1 CD19 0 FCGR3A 0 NCAM1 0 CD14 0,

CD3D 0 CD3E 0 CD4 0 CD8A 0 CD19 1 FCGR3A 0 NCAM1 0 CD14 0,

CD3D 0 CD3E 0 CD4 0 CD8A 0 CD19 0 FCGR3A 1 NCAM1 0 CD14 0,

CD3D 0 CD3E 0 CD4 0 CD8A 0 CD19 0 FCGR3A 0 NCAM1 1 CD14 0,

CD3D 0 CD3E 0 CD4 0 CD8A 0 CD19 0 FCGR3A 0 NCAM1 0 CD14 1;

* Analysis of pooled Study 1 and Study 2 samples. ;

* Characterizing pooled regressor matrix. ;

proc means data=regressors_z n nmiss mean stddev min max;

title 'Pooled sample: Descriptive statistics for final vertical data matrix';

var Study Male Age White BMI Alcohol Smoke Ill CD3D CD3E CD4 CD8A

CD19 FCGR3A NCAM1 CD14 Hedonic Eudaimonic PWB SWB Flourish TotalMHC

BrownPWB BrownSWB;

proc chart data=regressors_z;

hbar Male White Alcohol Smoke Flourish / discrete;

proc univariate data=regressors_z plot;

var Hedonic Eudaimonic PWB SWB TotalMHC BrownPWB BrownSWB;

proc print data=regressors_z uniform;

title 'Pooled sample: Final regressor matrix';

var Study PID Male Age White BMI Alcohol Smoke Ill CD3D CD3E CD4 CD8A

CD19 FCGR3A NCAM1 CD14 Hedonic Eudaimonic PWB SWB Flourish TotalMHC

BrownPWB BrownSWB;

proc reg data=regressors_z;

title 'Pooled sample: Regressor matrix characterization - Hedonic + Eudaimonic';

model Study = Male Age White BMI Alcohol Smoke Ill CD3D CD3E CD4 CD8A

CD19 FCGR3A NCAM1 CD14 Hedonic Eudaimonic / tol vif;

proc reg data=regressors_z;

title 'Pooled sample: Regressor matrix characterization - Hedonic + PWB + SWB';

model Study = Male Age White BMI Alcohol Smoke Ill CD3D CD3E CD4 CD8A

CD19 FCGR3A NCAM1 CD14 Hedonic PWB SWB / tol vif;

proc reg data=regressors_z;

title 'Pooled sample: Regressor matrix characterization - Flourish';

model Study = Male Age White BMI Alcohol Smoke Ill CD3D CD3E CD4 CD8A

CD19 FCGR3A NCAM1 CD14 Flourish / tol vif;

proc reg data=regressors_z;

title 'Pooled sample: Regressor matrix characterization - TotalMHC';

model Study = Male Age White BMI Alcohol Smoke Ill CD3D CD3E CD4 CD8A

CD19 FCGR3A NCAM1 CD14 TotalMHC / tol vif;

proc reg data=regressors_z;

title 'Pooled sample: Regressor matrix characterization - Hedonic + BrownPWB + BrownSWB';

model Study = Male Age White BMI Alcohol Smoke Ill CD3D CD3E CD4 CD8A

CD19 FCGR3A NCAM1 CD14 Hedonic BrownPWB BrownSWB / tol vif;

proc corr data=regressors_z pearson fisher;

title 'Pooled sample: Regressor matrix characterization - simple correlations';

var Hedonic Eudaimonic PWB SWB BrownPWB BrownSWB;

proc corr data=regressors_z pearson fisher;

title 'Pooled sample: Potential confounder characterization';

var Hedonic Eudaimonic PWB SWB Flourish TotalMHC BrownPWB BrownSWB;

with Male Age White BMI Alcohol Smoke Ill CD3D CD3E CD4 CD8A CD19 FCGR3A NCAM1 CD14;

* Characterize final stacked data matrix. ;

proc means data=pooled_z n nmiss mean stddev min max;

title 'Pooled sample: Descriptive statistics for final vertical data matrix';

var Study Male Age White BMI Alcohol Smoke Ill CD3D CD3E CD4 CD8A CD19

FCGR3A NCAM1 CD14 Hedonic Eudaimonic PWB SWB Flourish TotalMHC

BrownPWB BrownSWB;

* Assess heteroscedasticity. ;

proc glm data=vertical;

title 'Pooled sample: Brown-Forsythe test of heteroscedasticity across Genes';

class Gene;

model Expression = Gene;

means Gene / hovtest=bf;

proc glm data=vertical;

title 'Pooled sample: Brown-Forsythe test of heteroscedasticity across Study';

class Gene Study;

model Expression = Study;

means Study / hovtest=bf;

proc sort data=vertical;

by Study;

proc glm data=vertical;

title 'Study 1 and 2 separately: Brown-Forsythe test of heteroscedasticity across Genes';

class Gene;

model Expression = Gene;

means Gene / hovtest=bf;

by Study;

* Analyze data using PROC MIXED. ;

proc mixed data=pooled_z method=ml;

title 'Pooled sample - Model 1: Eudaimonic well-being + Hedonic well-being';

class Gene PID Study;

model Expression = Gene Study Male Age White BMI Ill Alcohol Smoke

CD3D CD3E CD4 CD8A CD19 FCGR3A NCAM1 CD14 Hedonic Eudaimonic / solution cl;

repeated Gene / subject=PID type=csh;

contrast 'LeukSubsets' CD3D 1 CD3E 0 CD4 0 CD8A 0 CD19 0 FCGR3A 0 NCAM1 0 CD14 0,

CD3D 0 CD3E 1 CD4 0 CD8A 0 CD19 0 FCGR3A 0 NCAM1 0 CD14 0,

CD3D 0 CD3E 0 CD4 1 CD8A 0 CD19 0 FCGR3A 0 NCAM1 0 CD14 0,

CD3D 0 CD3E 0 CD4 0 CD8A 1 CD19 0 FCGR3A 0 NCAM1 0 CD14 0,

CD3D 0 CD3E 0 CD4 0 CD8A 0 CD19 1 FCGR3A 0 NCAM1 0 CD14 0,

CD3D 0 CD3E 0 CD4 0 CD8A 0 CD19 0 FCGR3A 1 NCAM1 0 CD14 0,

CD3D 0 CD3E 0 CD4 0 CD8A 0 CD19 0 FCGR3A 0 NCAM1 1 CD14 0,

CD3D 0 CD3E 0 CD4 0 CD8A 0 CD19 0 FCGR3A 0 NCAM1 0 CD14 1;

contrast 'Well-being' Hedonic 0 Eudaimonic 1,

Hedonic 1 Eudaimonic 0;

estimate 'Eud-Hed' Eudaimonic 1 Hedonic -1;

proc mixed data=pooled_z method=ml;

title 'Pooled sample - Model 2: Hedonic well-being + Social well-being + Psychological well-being';

class Gene PID Study;

model Expression = Gene Study Male Age White BMI Ill Alcohol Smoke

CD3D CD3E CD4 CD8A CD19 FCGR3A NCAM1 CD14 Hedonic SWB PWB / solution cl;

repeated Gene / subject=PID type=csh;

contrast 'LeukSubsets' CD3D 1 CD3E 0 CD4 0 CD8A 0 CD19 0 FCGR3A 0 NCAM1 0 CD14 0,

CD3D 0 CD3E 1 CD4 0 CD8A 0 CD19 0 FCGR3A 0 NCAM1 0 CD14 0,

CD3D 0 CD3E 0 CD4 1 CD8A 0 CD19 0 FCGR3A 0 NCAM1 0 CD14 0,

CD3D 0 CD3E 0 CD4 0 CD8A 1 CD19 0 FCGR3A 0 NCAM1 0 CD14 0,

CD3D 0 CD3E 0 CD4 0 CD8A 0 CD19 1 FCGR3A 0 NCAM1 0 CD14 0,

CD3D 0 CD3E 0 CD4 0 CD8A 0 CD19 0 FCGR3A 1 NCAM1 0 CD14 0,

CD3D 0 CD3E 0 CD4 0 CD8A 0 CD19 0 FCGR3A 0 NCAM1 1 CD14 0,

CD3D 0 CD3E 0 CD4 0 CD8A 0 CD19 0 FCGR3A 0 NCAM1 0 CD14 1;

contrast 'Well-being' Hedonic 1 SWB 0 PWB 0,

Hedonic 0 SWB 1 PWB 0,

Hedonic 0 SWB 0 PWB 1;

contrast 'Eudaimonic' SWB 1 PWB 0,

SWB 0 PWB 1;

estimate 'SWB-PWB' SWB 1 PWB -1;

estimate 'SWB-Hedonic' Hedonic -1 SWB 1;

estimate 'PWB-Hedonic' Hedonic -1 PWB 1;

proc mixed data=pooled_z method=ml;

title 'Pooled sample - Model 3: Social well-being alone';

class Gene PID Study;

model Expression = Gene Study Male Age White BMI Ill Alcohol Smoke

CD3D CD3E CD4 CD8A CD19 FCGR3A NCAM1 CD14 SWB / solution cl;

repeated Gene / subject=PID type=csh;

contrast 'LeukSubsets' CD3D 1 CD3E 0 CD4 0 CD8A 0 CD19 0 FCGR3A 0 NCAM1 0 CD14 0,

CD3D 0 CD3E 1 CD4 0 CD8A 0 CD19 0 FCGR3A 0 NCAM1 0 CD14 0,

CD3D 0 CD3E 0 CD4 1 CD8A 0 CD19 0 FCGR3A 0 NCAM1 0 CD14 0,

CD3D 0 CD3E 0 CD4 0 CD8A 1 CD19 0 FCGR3A 0 NCAM1 0 CD14 0,

CD3D 0 CD3E 0 CD4 0 CD8A 0 CD19 1 FCGR3A 0 NCAM1 0 CD14 0,

CD3D 0 CD3E 0 CD4 0 CD8A 0 CD19 0 FCGR3A 1 NCAM1 0 CD14 0,

CD3D 0 CD3E 0 CD4 0 CD8A 0 CD19 0 FCGR3A 0 NCAM1 1 CD14 0,

CD3D 0 CD3E 0 CD4 0 CD8A 0 CD19 0 FCGR3A 0 NCAM1 0 CD14 1;

proc mixed data=pooled_z method=ml;

title 'Pooled sample - Model 4: Psychological well-being alone';

class Gene PID Study;

model Expression = Gene Study Male Age White BMI Ill Alcohol Smoke

CD3D CD3E CD4 CD8A CD19 FCGR3A NCAM1 CD14 PWB / solution cl;

repeated Gene / subject=PID type=csh;

contrast 'LeukSubsets' CD3D 1 CD3E 0 CD4 0 CD8A 0 CD19 0 FCGR3A 0 NCAM1 0 CD14 0,

CD3D 0 CD3E 1 CD4 0 CD8A 0 CD19 0 FCGR3A 0 NCAM1 0 CD14 0,

CD3D 0 CD3E 0 CD4 1 CD8A 0 CD19 0 FCGR3A 0 NCAM1 0 CD14 0,

CD3D 0 CD3E 0 CD4 0 CD8A 1 CD19 0 FCGR3A 0 NCAM1 0 CD14 0,

CD3D 0 CD3E 0 CD4 0 CD8A 0 CD19 1 FCGR3A 0 NCAM1 0 CD14 0,

CD3D 0 CD3E 0 CD4 0 CD8A 0 CD19 0 FCGR3A 1 NCAM1 0 CD14 0,

CD3D 0 CD3E 0 CD4 0 CD8A 0 CD19 0 FCGR3A 0 NCAM1 1 CD14 0,

CD3D 0 CD3E 0 CD4 0 CD8A 0 CD19 0 FCGR3A 0 NCAM1 0 CD14 1;

proc mixed data=pooled_z method=ml;

title 'Pooled sample - Model 5: Categorical Flourishing';

class Gene PID Study;

model Expression = Gene Study Male Age White BMI Ill Alcohol Smoke

CD3D CD3E CD4 CD8A CD19 FCGR3A NCAM1 CD14 Flourish / solution cl;

repeated Gene / subject=PID type=csh;

contrast 'LeukSubsets' CD3D 1 CD3E 0 CD4 0 CD8A 0 CD19 0 FCGR3A 0 NCAM1 0 CD14 0,

CD3D 0 CD3E 1 CD4 0 CD8A 0 CD19 0 FCGR3A 0 NCAM1 0 CD14 0,

CD3D 0 CD3E 0 CD4 1 CD8A 0 CD19 0 FCGR3A 0 NCAM1 0 CD14 0,

CD3D 0 CD3E 0 CD4 0 CD8A 1 CD19 0 FCGR3A 0 NCAM1 0 CD14 0,

CD3D 0 CD3E 0 CD4 0 CD8A 0 CD19 1 FCGR3A 0 NCAM1 0 CD14 0,

CD3D 0 CD3E 0 CD4 0 CD8A 0 CD19 0 FCGR3A 1 NCAM1 0 CD14 0,

CD3D 0 CD3E 0 CD4 0 CD8A 0 CD19 0 FCGR3A 0 NCAM1 1 CD14 0,

CD3D 0 CD3E 0 CD4 0 CD8A 0 CD19 0 FCGR3A 0 NCAM1 0 CD14 1;

proc mixed data=pooled_z method=ml;

title 'Pooled sample - Model 6: TotalMHC';

class Gene PID Study;

model Expression = Gene Study Male Age White BMI Ill Alcohol Smoke

CD3D CD3E CD4 CD8A CD19 FCGR3A NCAM1 CD14 TotalMHC / solution cl;

repeated Gene / subject=PID type=csh;

contrast 'LeukSubsets' CD3D 1 CD3E 0 CD4 0 CD8A 0 CD19 0 FCGR3A 0 NCAM1 0 CD14 0,

CD3D 0 CD3E 1 CD4 0 CD8A 0 CD19 0 FCGR3A 0 NCAM1 0 CD14 0,

CD3D 0 CD3E 0 CD4 1 CD8A 0 CD19 0 FCGR3A 0 NCAM1 0 CD14 0,

CD3D 0 CD3E 0 CD4 0 CD8A 1 CD19 0 FCGR3A 0 NCAM1 0 CD14 0,

CD3D 0 CD3E 0 CD4 0 CD8A 0 CD19 1 FCGR3A 0 NCAM1 0 CD14 0,

CD3D 0 CD3E 0 CD4 0 CD8A 0 CD19 0 FCGR3A 1 NCAM1 0 CD14 0,

CD3D 0 CD3E 0 CD4 0 CD8A 0 CD19 0 FCGR3A 0 NCAM1 1 CD14 0,

CD3D 0 CD3E 0 CD4 0 CD8A 0 CD19 0 FCGR3A 0 NCAM1 0 CD14 1;

proc mixed data=pooled_z method=ml;

title 'Pooled sample - Model 7: Hedonic well-being + BrownPsychological + BrownSocial';

class Gene PID Study;

model Expression = Gene Study Male Age White BMI Ill Alcohol Smoke

CD3D CD3E CD4 CD8A CD19 FCGR3A NCAM1 CD14 Hedonic BrownPWB BrownSWB / solution cl;

repeated Gene / subject=PID type=csh;

contrast 'LeukSubsets' CD3D 1 CD3E 0 CD4 0 CD8A 0 CD19 0 FCGR3A 0 NCAM1 0 CD14 0,

CD3D 0 CD3E 1 CD4 0 CD8A 0 CD19 0 FCGR3A 0 NCAM1 0 CD14 0,

CD3D 0 CD3E 0 CD4 1 CD8A 0 CD19 0 FCGR3A 0 NCAM1 0 CD14 0,

CD3D 0 CD3E 0 CD4 0 CD8A 1 CD19 0 FCGR3A 0 NCAM1 0 CD14 0,

CD3D 0 CD3E 0 CD4 0 CD8A 0 CD19 1 FCGR3A 0 NCAM1 0 CD14 0,

CD3D 0 CD3E 0 CD4 0 CD8A 0 CD19 0 FCGR3A 1 NCAM1 0 CD14 0,

CD3D 0 CD3E 0 CD4 0 CD8A 0 CD19 0 FCGR3A 0 NCAM1 1 CD14 0,

CD3D 0 CD3E 0 CD4 0 CD8A 0 CD19 0 FCGR3A 0 NCAM1 0 CD14 1;

contrast 'Well-being' Hedonic 1 BrownPWB 0 BrownSWB 0,

Hedonic 0 BrownPWB 1 BrownSWB 0,

Hedonic 0 BrownPWB 0 BrownSWB 1;

contrast 'Eudaimonic' BrownPWB 1 BrownSWB 0,

BrownPWB 0 BrownSWB 1;

estimate 'BrownPWB-BrownSWB' BrownPWB 1 BrownSWB -1;

estimate 'BrownPWB-Hedonic' Hedonic -1 BrownPWB 1;

estimate 'BrownSWB-Hedonic' Hedonic -1 BrownSWB 1;

proc mixed data=pooled_z method=ml;

title 'Pooled sample - Model 8: BrownPsychological alone';

class Gene PID Study;

model Expression = Gene Study Male Age White BMI Ill Alcohol Smoke

CD3D CD3E CD4 CD8A CD19 FCGR3A NCAM1 CD14 BrownPWB / solution cl;

repeated Gene / subject=PID type=csh;

contrast 'LeukSubsets' CD3D 1 CD3E 0 CD4 0 CD8A 0 CD19 0 FCGR3A 0 NCAM1 0 CD14 0,

CD3D 0 CD3E 1 CD4 0 CD8A 0 CD19 0 FCGR3A 0 NCAM1 0 CD14 0,

CD3D 0 CD3E 0 CD4 1 CD8A 0 CD19 0 FCGR3A 0 NCAM1 0 CD14 0,

CD3D 0 CD3E 0 CD4 0 CD8A 1 CD19 0 FCGR3A 0 NCAM1 0 CD14 0,

CD3D 0 CD3E 0 CD4 0 CD8A 0 CD19 1 FCGR3A 0 NCAM1 0 CD14 0,

CD3D 0 CD3E 0 CD4 0 CD8A 0 CD19 0 FCGR3A 1 NCAM1 0 CD14 0,

CD3D 0 CD3E 0 CD4 0 CD8A 0 CD19 0 FCGR3A 0 NCAM1 1 CD14 0,

CD3D 0 CD3E 0 CD4 0 CD8A 0 CD19 0 FCGR3A 0 NCAM1 0 CD14 1;

proc mixed data=pooled_z method=ml;

title 'Pooled sample - Model 9: BrownSocial alone';

class Gene PID Study;

model Expression = Gene Study Male Age White BMI Ill Alcohol Smoke

CD3D CD3E CD4 CD8A CD19 FCGR3A NCAM1 CD14 BrownSWB / solution cl;

repeated Gene / subject=PID type=csh;

contrast 'LeukSubsets' CD3D 1 CD3E 0 CD4 0 CD8A 0 CD19 0 FCGR3A 0 NCAM1 0 CD14 0,

CD3D 0 CD3E 1 CD4 0 CD8A 0 CD19 0 FCGR3A 0 NCAM1 0 CD14 0,

CD3D 0 CD3E 0 CD4 1 CD8A 0 CD19 0 FCGR3A 0 NCAM1 0 CD14 0,

CD3D 0 CD3E 0 CD4 0 CD8A 1 CD19 0 FCGR3A 0 NCAM1 0 CD14 0,

CD3D 0 CD3E 0 CD4 0 CD8A 0 CD19 1 FCGR3A 0 NCAM1 0 CD14 0,

CD3D 0 CD3E 0 CD4 0 CD8A 0 CD19 0 FCGR3A 1 NCAM1 0 CD14 0,

CD3D 0 CD3E 0 CD4 0 CD8A 0 CD19 0 FCGR3A 0 NCAM1 1 CD14 0,

CD3D 0 CD3E 0 CD4 0 CD8A 0 CD19 0 FCGR3A 0 NCAM1 0 CD14 1;
