# Supplementary material for: Psychological Well-Being and the Human Conserved Transcriptional Response to Adversity
Source: PLoS One. 2015 Mar 26;10(3):e0121839. doi: 10.1371/journal.pone.0121839 (PMC4374902; doi:10.1371/journal.pone.0121839)
Supplement: S1 Table — (DOC) [file pone.0121839.s004.doc]

**Table S1 – Confirmatory Factor Analysis of alternative MHC-SF representations of well-being**

|  | 1-d Total well-being | 2-d Hedonic Eudaimonic | | | 3-d Hedonic Social Psychological | | Alternative 2-d General Social | | Alternative 3-d Hedonic Social Psychological | |
| --- | --- | --- | --- | --- | --- | --- | --- | --- | --- | --- |
| **A. Model fit statistics^1^** | | | | | | | | | | |
| Factors | 1 | | 2 | | | 3 | 2 | | 3 | |
| Model parameters | 28 | | 29 | | | 31 | 29 | | 31 | |
| X^2^ | 209.99 | | 170.96 | | | 162.41 | 186.03 | | 148.98 | |
| df | 77 | | 76 | | | 74 | 76 | | 74 | |
| AIC | 265.99 | | 228.96 | | | 224.41 | 244.03 | | 210.98 | |
| AGFI | .7342 | | .7809 | | | .7829 | .7497 | | .7960 | |
| BCFI | .8732 | | .9095 | | | .9157 | .8951 | | .9285 | |
| **B. Item loadings^2^** | | | | | | | | | | |
| Item 1 | .71 ± .05 (14.47) | | | .84 ± .03 (24.09)^h^ | .84 ± .03 (24.02)^h^ | | .71 ± .05 (14.81)^g^ | | | .84 ± .04 (23.94)^h^ |
| Item 2 | .73 ± .05 (16.07) | | | .85 ± .03 (24.84)^h^ | .85 ± .03 (24.83)^h^ | | .74 ± .04 (16.77)^g^ | | | .85 ± .03 (25.56)^h^ |
| Item 3 | .79 ± .04 (21.45) | | | .82 ± .04 (21.78)^h^ | .82 ± .04 (21.91)^h^ | | .80 ± .04 (21.83)^g^ | | | .82 ± .04 (21.60)^h^ |
| Item 4 | .83 ± .03 (25.59) | | | .83 ± .03 (25.15)^e^ | .86 ± .03 (27.00)^s^ | | .83 ± .03 (25.47)^g^ | | | .83 ± .03 (25.32)^p^ |
| Item 5 | .69 ± .05 (13.75) | | | .69± .05 (13.74)^e^ | .72 ± .05 (14.67)^s^ | | .70 ± .05 (13.89)^g^ | | | .70 ± .05 (13.94)^p^ |
| Item 6 | .57 ± .06 ( 8.78) | | | .56 ± .07 ( 8.55)^e^ | .60 ± .06 ( 9.59)^s^ | | .72 ± .06 (12.38)^s^ | | | .69 ± .06 (11.35)^s^ |
| Item 7 | .62 ± .06 (10.54) | | | .65 ± .06 (11.54)^e^ | .65 ± .06 (11.06)^s^ | | .70 ± .06 (11.85)^s^ | | | .74 ± .06 (13.20)^s^ |
| Item 8 | .57 ± .06 ( 8.81) | | | .58 ± .06 ( 9.01)^e^ | .59 ± .06 ( 9.17)^s^ | | .73 ± .06 (12.89)^s^ | | | .72 ± .06 (12.46)^s^ |
| Item 9 | .79 ± .04 (21.57) | | | .81 ± .03 (23.39)^e^ | .82 ± .03 (23.97)^p^ | | .79 ± .04 (21.14)^g^ | | | .81 ± .04 (23.04)^p^ |
| Item 10 | .78 ± .04 (20.19) | | | .78 ± .04 (20.09)^e^ | .79 ± .04 (20.78)^p^ | | .78 ± .04 (19.92)^g^ | | | .78 ± .04 (20.00)^p^ |
| Item 11 | .78 ± .04 (20.33) | | | .78 ± .04 (19.55)^e^ | .79 ± .04 (20.12)^p^ | | .79 ± .04 (20.80)^g^ | | | .78 ± .04 (20.10)^p^ |
| Item 12 | .66 ± .05 (11.97) | | | .66 ± .05 (12.27)^e^ | .66 ± .05 (12.09)^p^ | | .65 ± .06 (11.88)^g^ | | | .66 ± .05 (12.25)^p^ |
| Item 13 | .77 ± .04 (19.21) | | | .78 ± .04 (19.80)^e^ | .79 ± .04 (20.57)^p^ | | .77 ± .04 (19.09)^g^ | | | .78 ± .04 (19.77)^p^ |
| Item 14 | .73 ± .05 (16.12) | | | .74 ± .04 (16.59)^e^ | .74 ± .05 (16.27)^p^ | | .73 ± .05 (15.92)^g^ | .74 ± .05 (16.49)^p^ | | |
| **C. Factor correlations^3^** | | | | | | | | | | |
| F1 vs F2 | n/a | | .83 ± .04 (21.25) | | .81 ± .05 (16.68) | | .78 ± .06 (14.22) | | | .63 ± .08 ( 8.09) |
| F1 vs F3 | n/a | | n/a | | .83 ± .04 (19.69) | | n/a | | | .84 ± .04 (22.13) |
| F2 vs F3 | n/a | | n/a | | .93 ± .03 (31.18) | | n/a | | | .81 ± .05 (15.32) |

1. Analyses based on n = 121 with complete data on 14 MHC-SF items. AIC = Akaike Information Criterion, AGFI = Adjusted Goodness of Fit Index, BCFI = Bentler Comparative Fit Index

2. Standardized loading coefficients ± standard error (*t*-statistic). Items are ordered as in **S7 File Supplemental Materials and Methods: Confirmatory Factor Analysis** and the MHC-SF instrument. Horizontal bars delineate factor-item allocations for each model.

3. Standardized covariance ± standard error (*t*-statistic)

Lettered footnotes indicate item-factor allocation: h = hedonic, e = eudaimonic, s = social, p = psychological, g = general
