# Supplementary material for: Psychological Well-Being and the Human Conserved Transcriptional Response to Adversity
Source: PLoS One. 2015 Mar 26;10(3):e0121839. doi: 10.1371/journal.pone.0121839 (PMC4374902; doi:10.1371/journal.pone.0121839)
Supplement: S2 Table — (DOC) [file pone.0121839.s005.doc]

**Table S2 – Confirmation study association of well-being with gene expression controlling for depressive symptoms**

|  | Well-being dimension | Association *b* ± SE^1^ | | Test statistic | | *p*-value | |  |
| --- | --- | --- | --- | --- | --- | --- | --- | --- |
| **A. 2-dimensional** |  |  | |  | |  | |  |
|  | Hedonic well-being | -0.0480 ± 0.1246 | | *t*(102) = -0.38 | | .7012 | |  |
|  | Eudaimonic well-being | -0.5836 ± 0.1267 | | *t*(102) = -4.61 | | < .0001 | |  |
|  |  |  | |  | |  | |  |
| **B. 3-dimensional** |  |  | |  | |  | |  |
|  | Hedonic well-being | -0.0358 ± 0.1248 | | *t*(101) = -0.29 | | .7747 | |  |
|  | Psychological well-being | -0.4428 ± 0.1568 | | *t*(101) = -2.82 | | .0057 | |  |
|  | Social well-being | -0.1861 ± 0.1364 | | *t*(101) = -1.36 | | .1755 | |  |
|  |  |  | |  | |  | |  |
| **C. 1-dimensional** |  |  | |  | |  | |  |
|  | Total well-being | -0.6275 ± 0.0976 | | *t*(103) = -6.43 | | < .0001 | |  |
|  |  |  | |  | |  | |  |
| **D. Categorical** |  |  | |  | |  | |  |
|  | Flourishing mental health | -0.8289 ± 0.1832 | | *t*(103) = -4.53 | | < .0001 | |  |
|  |  | |  | |  | |  | |

1. Partial regression coefficients relating standardized gene expression values to standardized scores on 1-, 2-, and 3-d representations of well-being (A, B, C) or a categorical representation of flourishing mental health (D). All associations are adjusted for age, sex, race, BMI, smoking, alcohol consumption, illness symptoms, gene transcript covariates marking major leukocyte subsets, and depressive symptoms as measured by the Center for Epidemiologic Studies Depression scale.
