# Supplementary material for: Psychological Well-Being and the Human Conserved Transcriptional Response to Adversity
Source: PLoS One. 2015 Mar 26;10(3):e0121839. doi: 10.1371/journal.pone.0121839 (PMC4374902; doi:10.1371/journal.pone.0121839)
Supplement: S3 Table — (DOC) [file pone.0121839.s006.doc]

**Table S3 – Confirmation study association of well-being with gene expression: Unstructured covariance matrix**

|  | Well-being dimension | Association *b* ± SE^1^ | Test statistic | | *p*-value | |  |
| --- | --- | --- | --- | --- | --- | --- | --- |
| **Maximum likelihood estimation** | | | | | | |  |
| **A. 2-dimensional** |  |  |  | |  | |  |
|  | Hedonic well-being | -0.0141 ± 0.0103 | *t*(104) = -1.38 | | .1701 | |  |
|  | Eudaimonic well-being | -0.0026 ± 0.0105 | *t*(104) = -0.25 | | .8067 | |  |
|  |  |  |  | |  | |  |
| **B. 3-dimensional** |  |  |  | |  | |  |
|  | Hedonic well-being | -0.0080 ± 0.0101 | *t*(103) = -0.80 | | .4242 | |  |
|  | Psychological well-being | -0.0429 ± 0.0127 | *t*(103) = -3.39 | | .0010 | |  |
|  | Social well-being | 0.0350 ± 0.0113 | *t*(103) = 3.10 | | .0025 | |  |
|  |  |  |  | |  | |  |
| **C. 1-dimensional** |  |  |  | |  | |  |
|  | Total well-being | -0.0145 ± 0.0071 | *t*(105) = -2.05 | | .0432 | |  |
|  |  |  |  | |  | |  |
| **D. Categorical** |  |  |  | |  | |  |
|  | Flourishing mental health | -0.0114 ± 0.0143 | *t*(105) = -0.79 | | .4290 | |  |
| **Restricted maximum likelihood estimation** | | | | | | | |
| **E. 2-dimensional** |  |  |  |  | |  | |
|  | Hedonic well-being | Not available^2^ | | | | |  |
|  | Eudaimonic well-being | Not available^2^ | | | | |  |
|  |  |  |  | |  | |  |
| **F. 3-dimensional** |  |  |  | |  | |  |
|  | Hedonic well-being | -0.0080 ± 0.0109 | *t*(103) = -0.73 | | .4665 | |  |
|  | Psychological well-being | -0.0429 ± 0.0138 | *t*(103) = -3.11 | | .0024 | |  |
|  | Social well-being | 0.0350 ± 0.0123 | *t*(103) = 2.85 | | .0053 | |  |
|  |  |  |  | |  | |  |
| **G. 1-dimensional** |  |  |  | |  | |  |
|  | Total well-being | Not available^2^ | | | | |  |
|  |  |  |  | |  | |  |
| **H. Categorical** |  |  |  | |  | |  |
|  | Flourishing mental health | Not available^2^ | | | | |  |
|  |  |  |  | |  | |  |

**Minimum variance quadratic unbiased estimation**

| **I. 2-dimensional** |  |  | |  | |  | |  |
| --- | --- | --- | --- | --- | --- | --- | --- | --- |
|  | Hedonic well-being | | 0.0056 ± 0.0064 | | *t*(104) = 0.88 | | .3833 | |
|  | Eudaimonic well-being | | -0.0234 ± 0.0065 | | *t*(104) = -3.56 | | .0006 | |
|  |  | |  | |  | |  | |
| **J. 3-dimensional** |  | |  | |  | |  | |
|  | Hedonic well-being | | 0.0096 ± 0.0034 | | *t*(103) = 2.83 | | .0056 | |
|  | Psychological well-being | | -0.0537 ± 0.0043 | | *t*(103) = -12.51 | | < .0001 | |
|  | Social well-being | | 0.0255 ± 0.0038 | | *t*(103) = 6.66 | | < .0001 | |
|  |  | |  | |  | |  | |
| **K. 1-dimensional** |  | |  | |  | |  | |
|  | Total well-being | | -0.0192 ± 0.0033 | | *t*(105) = -5.79 | | < .0001 | |
|  |  | |  | |  | |  | |
| **L. Categorical** |  | |  | |  | |  | |
|  | Flourishing mental health | | -0.0059 ± 0.0074 | | *t*(105) = -0.81 | | .4220 | |
|  |  | |  | |  | |  | |

1. Partial regression coefficients relating standardized gene expression values to standardized scores on 1-, 2-, and 3-d representations of well-being (A, B, C) or a categorical representation of flourishing mental health (D). All associations are adjusted for age, sex, race, BMI, smoking, alcohol consumption, illness symptoms, and gene transcript covariates marking major leukocyte subsets.

2. Estimation algorithm failed to converge.
