# Supplementary material for: Psychological Well-Being and the Human Conserved Transcriptional Response to Adversity
Source: PLoS One. 2015 Mar 26;10(3):e0121839. doi: 10.1371/journal.pone.0121839 (PMC4374902; doi:10.1371/journal.pone.0121839)
Supplement: S4 Table — (DOC) [file pone.0121839.s007.doc]

**Table S4 – Association of alternative MHC-SF well-being measures with gene expression**

|  | Well-being dimension | Association *b* ± SE^1^ | Test statistic | *p*-value | VIF^2^ |
| --- | --- | --- | --- | --- | --- |
| **A. Confirmation sample** | | | | | |
| Hedonic well-being | | 0.060 ± 0.124 | *t*(103) = 0.48 | .6314 | 2.58 |
| Alternative psychological well-being | | -0.277 ± 0.149 | *t*(103) = -1.85 | .0665 | 3.72 |
| Alternative social well-being | | -0.264 ± 0.113 | *t*(103) = -2.34 | .0211 | 2.12 |
| **B. Pooled sample** | | | | | |
| Hedonic well-being | | 0.032 ± 0.043 | *t*(178) = 0.74 | .4589 | 2.66 |
| Alternative psychological well-being | | 0.032 ± 0.049 | *t*(178) = 0.65 | .5173 | 3.39 |
| Alternative social well-being | | -0.144 ± 0.035 | *t*(178) = -4.17 | < .0001 | 1.68 |

1. Partial regression coefficients relating standardized gene expression values to standardized scores on the alternative 3-d representation of well-being in analyses of the confirmation sample (A.) and pooled discovery and confirmation samples (B.). All associations are adjusted for age, sex, race, BMI, smoking, alcohol consumption, illness symptoms, and gene transcript covariates marking major leukocyte subsets.

2. Variance Inflation Factor. Values > 10 indicate significant multicollinearity.
