# Supplementary material for: Psychological Well-Being and the Human Conserved Transcriptional Response to Adversity
Source: PLoS One. 2015 Mar 26;10(3):e0121839. doi: 10.1371/journal.pone.0121839 (PMC4374902; doi:10.1371/journal.pone.0121839)
Supplement: S5 Table — (DOC) [file pone.0121839.s008.doc]

**Table S5 – Confirmation study association of well-being with gene expression: Including imputed *IL6* data**

|  | Well-being dimension | Association *b* ± SE^1^ | Test statistic | *p*-value |
| --- | --- | --- | --- | --- |
| **A. 2-dimensional** |  |  |  |  |
|  | Hedonic well-being | 0.062 ± 0.125 | *t*(104) = 0.50 | .6204 |
|  | Eudaimonic well-being | -0.501 ± 0.128 | *t*(104) = -3.92 | .0002 |
|  |  |  |  |  |
| **B. 3-dimensional** |  |  |  |  |
|  | Hedonic well-being | 0.074 ± 0.125 | *t*(103) = 0.59 | .5538 |
|  | Psychological well-being | -0.378 ± 0.158 | *t*(103) = -2.40 | .0183 |
|  | Social well-being | -0.161 ± 0.141 | *t*(103) = -1.14 | .2553 |
|  |  |  |  |  |
| **C. 1-dimensional** |  |  |  |  |
|  | Total well-being | -0.450 ± 0.088 | *t*(105) = -5.12 | < .0001 |
|  |  |  |  |  |
| **D. Categorical** |  |  |  |  |
|  | Flourishing mental health | -0.631 ± 0.180 | *t*(105) = -3.51 | .0007 |
|  |  |  |  |  |

1. Partial regression coefficients relating standardized gene expression values to standardized scores on 1-, 2-, and 3-d representations of well-being (A, B, C) or a categorical representation of flourishing mental health (D). All associations are adjusted for age, sex, race, BMI, smoking, alcohol consumption, illness symptoms, and gene transcript covariates marking major leukocyte subsets.
