# Supplementary material for: Correction: Psychological Well-Being and the Human Conserved Transcriptional Response to Adversity
Source: PLoS One. 2016 Jun 3;11(6):e0157116. doi: 10.1371/journal.pone.0157116 (PMC4892531; doi:10.1371/journal.pone.0157116)
Supplement: S3 File — (PDF) [file pone.0157116.s001.pdf]

SAS Statistical Output:

Discovery Study: Eudaimonic & Hedonic well-being 1
Corrected GSE45330_Regressors

Obs	Variable	PctDif Per4SD
1	Eudaimonic	-7.20967
2	Hedonic	3.57777

Original GSE45330_Regressors (with coding error SOBC1_1299 White=4)

Obs	Variable	PctDif Per4SD
1	Eudaimonic	-7.36812
2	Hedonic	8.02316

Obs	Variable	PctDif Per4SD
1	Eudaimonic	-5.64916
2	Hedonic	7.15821

SAS Statistical Syntax:

```
options linesize=80;

* Reading Discovery Study regressors from space-delimited text file with header removed and PID trimmed to 10 characters. ;

data regressors_study1;
     infile 'GSE45330_Regressors.txt';
     input PID $ CHAR10. ZHedonic ZEudaimonic Hedonic Eudaimonic SWB PWB
     TotalMHC Flourish BrownPWB BrownSWB Male Age White BMI Alcohol Smoke
     Ill CD3D CD3E CD4 CD8A CD19 FCGR3A NCAM1 CD14;
     Study = 1;
proc sort;
     by PID;


* Reading Confirmation Study regressors from space-delimited text file with header removed and PID trimmed to 10 characters. ;

data regressors_study2;
     infile 'GSE55762_Regressors.txt';
     input PID $ CHAR10. Hedonic Eudaimonic SWB PWB TotalMHC Flourish
     BrownPWB BrownSWB Age White Male BMI Ill Smoke Alcohol CESD CD3E CD3D CD19
     CD4 CD8A FCGR3A NCAM1 CD14;
     Study = 2;
proc sort;
     by PID;



* Forming pooled regressor matrix. ;

data regressors;
     set regressors_study1 regressors_study2;

     * Deleting cases with missing covariate data. ;

     if Male = . then delete;
     if Age = . then delete;
     if White = . then delete;
     if BMI = . then delete;
     if Smoke = . then delete;
     if Alcohol = . then delete;
     if Ill = . then delete;



* Reading Discovery Study gene expression data from space-delimited text file. ;

data genes_study1;
     infile 'GSE45330_NormalizedExpression.txt';
     input Gene $ SOBC1_1283 SOBC1_1252 SOBC1_1235 SOBC1_1285 SOBC1_1255
     SOBC1_1601 SOBC1_1199 SOBC1_1480 SOBC1_1259 SOBC1_1256 SOBC1_1271
     SOBC1_1220 SOBC1_1225 SOBC1_1194 SOBC1_1333 SOBC1_1192 SOBC1_1260
     SOBC1_1188 SOBC1_1196 SOBC1_1186 SOBC1_1326 SOBC1_1277 SOBC1_1231
     SOBC1_1195 SOBC1_1197 SOBC1_1300 SOBC1_1232 SOBC1_1441 SOBC1_1239
     SOBC1_1264 SOBC1_1185 SOBC1_1262 SOBC1_1306 SOBC1_1345 SOBC1_1343
     SOBC1_1297 SOBC1_1329 SOBC1_1245 SOBC1_1293 SOBC1_1189 SOBC1_1241
     SOBC1_1292 SOBC1_1328 SOBC1_1228 SOBC1_1294 SOBC1_1288 SOBC1_1580
     SOBC1_1422 SOBC1_1681 SOBC1_1344 SOBC1_1273 SOBC1_1223 SOBC1_1720
     SOBC1_1721 SOBC1_1287 SOBC1_1237 SOBC1_1440 SOBC1_1286 SOBC1_1740
     SOBC1_1760 SOBC1_1900 SOBC1_1247 SOBC1_1190 SOBC1_1201 SOBC1_1305
     SOBC1_1193 SOBC1_1246 SOBC1_1182 SOBC1_1263 SOBC1_1234 SOBC1_1198
     SOBC1_1299 SOBC1_1680 SOBC1_1600 SOBC1_1722 SOBC1_1683 SOBC1_1684
     SOBC1_1289 SOBC1_1880;

     * Removing data for genes not used. ;

     keep = 0;

     if gene = 'FOS'    then keep = 1;
     if gene = 'FOSB'   then keep = 1;
     if gene = 'FOSL1'  then keep = 1;
     if gene = 'FOSL2'  then keep = 1;
     if gene = 'GBP1'   then keep = 1;
     if gene = 'IFI16'  then keep = 1;
     if gene = 'IFI27'  then keep = 1;
     if gene = 'IFI27L1' then keep = 1;
     if gene = 'IFI27L2' then keep = 1;
     if gene = 'IFI30'  then keep = 1;
     if gene = 'IFI35'  then keep = 1;
     if gene = 'IFI44'  then keep = 1;
```

```
     if gene = 'IFI44L' then keep = 1;
     if gene = 'IFI6'   then keep = 1;
     if gene = 'IFIH1'  then keep = 1;
     if gene = 'IFIT1'  then keep = 1;
     if gene = 'IFIT1L' then keep = 1;
     if gene = 'IFIT2'  then keep = 1;
     if gene = 'IFIT3'  then keep = 1;
     if gene = 'IFIT5'  then keep = 1;
     if gene = 'IFITM1' then keep = 1;
     if gene = 'IFITM2' then keep = 1;
     if gene = 'IFITM3' then keep = 1;
     if gene = 'IFITM4P' then keep = 1;
     if gene = 'IFITM5' then keep = 1;
     if gene = 'IFNB1'  then keep = 1;
     if gene = 'IGJ'    then keep = 1;
     if gene = 'IGLL1'  then keep = 1;
     if gene = 'IGLL3'  then keep = 1;
     if gene = 'IL1A'   then keep = 1;
     if gene = 'IL1B'   then keep = 1;
     if gene = 'IL6'    then keep = 1;
     if gene = 'IL8'    then keep = 1;
     if gene = 'IRF2'   then keep = 1;
     if gene = 'IRF7'   then keep = 1;
     if gene = 'IRF8'   then keep = 1;
     if gene = 'JUN'    then keep = 1;
     if gene = 'JUNB'   then keep = 1;
     if gene = 'JUND'   then keep = 1;
     if gene = 'MX1'    then keep = 1;
     if gene = 'MX2'    then keep = 1;
     if gene = 'NFKB1'  then keep = 1;
     if gene = 'NFKB2'  then keep = 1;
     if gene = 'OAS1'   then keep = 1;
     if gene = 'OAS2'   then keep = 1;
     if gene = 'OAS3'   then keep = 1;
     if gene = 'OASL'   then keep = 1;
     if gene = 'PTGS1'  then keep = 1;
     if gene = 'PTGS2'  then keep = 1;
     if gene = 'REL'    then keep = 1;
     if gene = 'RELA'   then keep = 1;
     if gene = 'RELB'   then keep = 1;
     if gene = 'TNF'    then keep = 1;

     if keep ne 1 then delete;

proc sort;
     by gene;



* Reading Confirmation Study gene expression data from space-delimited text file. ;

data genes_study2;
     infile 'GSE55762_NormalizedExpression.txt';
     input Gene $ SOBC2_2112 SOBC2_2037 SOBC2_2072 SOBC2_2147 SOBC2_2076
     SOBC2_2093 SOBC2_2020 SOBC2_2121 SOBC2_2048 SOBC2_2073 SOBC2_2023
     SOBC2_2086 SOBC2_2202 SOBC2_2091 SOBC2_2104 SOBC2_2074 SOBC2_2044
     SOBC2_2089 SOBC2_2028 SOBC2_2144 SOBC2_2180 SOBC2_2123 SOBC2_2036
     SOBC2_2113 SOBC2_2187 SOBC2_2047 SOBC2_2168 SOBC2_2096 SOBC2_2042
     SOBC2_2166 SOBC2_2021 SOBC2_2031 SOBC2_2030 SOBC2_2167 SOBC2_2039
     SOBC2_2032 SOBC2_2119 SOBC2_2049 SOBC2_2171 SOBC2_2169 SOBC2_2026
     SOBC2_2131 SOBC2_2145 SOBC2_2024 SOBC2_2106 SOBC2_2201 SOBC2_2183
     SOBC2_2064 SOBC2_2092 SOBC2_2035 SOBC2_2043 SOBC2_2182 SOBC2_2061
     SOBC2_2079 SOBC2_2025 SOBC2_2136 SOBC2_2066 SOBC2_2639 SOBC2_2597
     SOBC2_2593 SOBC2_2603 SOBC2_2566 SOBC2_2637 SOBC2_2586 SOBC2_2592
     SOBC2_2629 SOBC2_2569 SOBC2_2567 SOBC2_2625 SOBC2_2607 SOBC2_2613
     SOBC2_2574 SOBC2_2605 SOBC2_2663 SOBC2_2647 SOBC2_2564 SOBC2_2599
     SOBC2_2575 SOBC2_2661 SOBC2_2675 SOBC2_2560 SOBC2_2638 SOBC2_2636
     SOBC2_2654 SOBC2_2579 SOBC2_2619 SOBC2_2659 SOBC2_2610 SOBC2_2623
     SOBC2_2606 SOBC2_2601 SOBC2_2626 SOBC2_2618 SOBC2_2561 SOBC2_2644
     SOBC2_2635 SOBC2_2550 SOBC2_2602 SOBC2_2554 SOBC2_2616 SOBC2_2648
     SOBC2_2594 SOBC2_2630 SOBC2_2622 SOBC2_2736 SOBC2_2609 SOBC2_2627
     SOBC2_2728 SOBC2_2632 SOBC2_2587 SOBC2_2596 SOBC2_2723 SOBC2_2714
     SOBC2_2642 SOBC2_2176 SOBC2_2186 SOBC2_2108 SOBC2_2165 SOBC2_2174
     SOBC2_2205 SOBC2_2097 SOBC2_2094 ;

     * Removing data for genes not used. ;

     keep = 0;

     if gene = 'FOS'    then keep = 1;
     if gene = 'FOSB'   then keep = 1;
```

```
     if gene = 'FOSL1'  then keep = 1;
     if gene = 'FOSL2'  then keep = 1;
     if gene = 'GBP1'   then keep = 1;
     if gene = 'IFI16'  then keep = 1;
     if gene = 'IFI27'  then keep = 1;
     if gene = 'IFI27L1' then keep = 1;
     if gene = 'IFI27L2' then keep = 1;
     if gene = 'IFI30'  then keep = 1;
     if gene = 'IFI35'  then keep = 1;
     if gene = 'IFI44'  then keep = 1;
     if gene = 'IFI44L' then keep = 1;
     if gene = 'IFI6'   then keep = 1;
     if gene = 'IFIH1'  then keep = 1;
     if gene = 'IFIT1'  then keep = 1;
     if gene = 'IFIT1L' then keep = 1;
     if gene = 'IFIT2'  then keep = 1;
     if gene = 'IFIT3'  then keep = 1;
     if gene = 'IFIT5'  then keep = 1;
     if gene = 'IFITM1' then keep = 1;
     if gene = 'IFITM2' then keep = 1;
     if gene = 'IFITM3' then keep = 1;
     if gene = 'IFITM4P' then keep = 1;
     if gene = 'IFITM5' then keep = 1;
     if gene = 'IFNB1'  then keep = 1;
     if gene = 'IGJ'    then keep = 1;
     if gene = 'IGLL1'  then keep = 1;
     if gene = 'IGLL3'  then keep = 1;
     if gene = 'IL1A'   then keep = 1;
     if gene = 'IL1B'   then keep = 1;
     if gene = 'IL6'    then keep = 1;
     if gene = 'IL8'    then keep = 1;
     if gene = 'IRF2'   then keep = 1;
     if gene = 'IRF7'   then keep = 1;
     if gene = 'IRF8'   then keep = 1;
     if gene = 'JUN'    then keep = 1;
     if gene = 'JUNB'   then keep = 1;
     if gene = 'JUND'   then keep = 1;
     if gene = 'MX1'    then keep = 1;
     if gene = 'MX2'    then keep = 1;
     if gene = 'NFKB1'  then keep = 1;
     if gene = 'NFKB2'  then keep = 1;
     if gene = 'OAS1'   then keep = 1;
     if gene = 'OAS2'   then keep = 1;
     if gene = 'OAS3'   then keep = 1;
     if gene = 'OASL'   then keep = 1;
     if gene = 'PTGS1'  then keep = 1;
     if gene = 'PTGS2'  then keep = 1;
     if gene = 'REL'    then keep = 1;
     if gene = 'RELA'   then keep = 1;
     if gene = 'RELB'   then keep = 1;
     if gene = 'TNF'    then keep = 1;

     if keep ne 1 then delete;

proc sort;
     by gene;



* Forming pooled gene expression matrix. ;

data genes;
     merge genes_study1 genes_study2;
     by gene;


* Transposing gene expression matrix to PID x Gene format. ;

proc transpose data=genes name = PID out=genes_horiz;
     id Gene;
     var SOBC1_1283 SOBC1_1252 SOBC1_1235 SOBC1_1285 SOBC1_1255 SOBC1_1601
     SOBC1_1199 SOBC1_1480 SOBC1_1259 SOBC1_1256 SOBC1_1271 SOBC1_1220
     SOBC1_1225 SOBC1_1194 SOBC1_1333 SOBC1_1192 SOBC1_1260 SOBC1_1188
     SOBC1_1196 SOBC1_1186 SOBC1_1326 SOBC1_1277 SOBC1_1231 SOBC1_1195
     SOBC1_1197 SOBC1_1300 SOBC1_1232 SOBC1_1441 SOBC1_1239 SOBC1_1264
     SOBC1_1185 SOBC1_1262 SOBC1_1306 SOBC1_1345 SOBC1_1343 SOBC1_1297
     SOBC1_1329 SOBC1_1245 SOBC1_1293 SOBC1_1189 SOBC1_1241 SOBC1_1292
     SOBC1_1328 SOBC1_1228 SOBC1_1294 SOBC1_1288 SOBC1_1580 SOBC1_1422
     SOBC1_1681 SOBC1_1344 SOBC1_1273 SOBC1_1223 SOBC1_1720 SOBC1_1721
     SOBC1_1287 SOBC1_1237 SOBC1_1440 SOBC1_1286 SOBC1_1740 SOBC1_1760
     SOBC1_1900 SOBC1_1247 SOBC1_1190 SOBC1_1201 SOBC1_1305 SOBC1_1193
```

```
     SOBC1_1246 SOBC1_1182 SOBC1_1263 SOBC1_1234 SOBC1_1198 SOBC1_1299
     SOBC1_1680 SOBC1_1600 SOBC1_1722 SOBC1_1683 SOBC1_1684 SOBC1_1289
     SOBC1_1880 SOBC2_2112 SOBC2_2037 SOBC2_2072 SOBC2_2147 SOBC2_2076
     SOBC2_2093 SOBC2_2020 SOBC2_2121 SOBC2_2048 SOBC2_2073 SOBC2_2023
     SOBC2_2086 SOBC2_2202 SOBC2_2091 SOBC2_2104 SOBC2_2074 SOBC2_2044
     SOBC2_2089 SOBC2_2028 SOBC2_2144 SOBC2_2180 SOBC2_2123 SOBC2_2036
     SOBC2_2113 SOBC2_2187 SOBC2_2047 SOBC2_2168 SOBC2_2096 SOBC2_2042
     SOBC2_2166 SOBC2_2021 SOBC2_2031 SOBC2_2030 SOBC2_2167 SOBC2_2039
     SOBC2_2032 SOBC2_2119 SOBC2_2049 SOBC2_2171 SOBC2_2169 SOBC2_2026
     SOBC2_2131 SOBC2_2145 SOBC2_2024 SOBC2_2106 SOBC2_2201 SOBC2_2183
     SOBC2_2064 SOBC2_2092 SOBC2_2035 SOBC2_2043 SOBC2_2182 SOBC2_2061
     SOBC2_2079 SOBC2_2025 SOBC2_2136 SOBC2_2066 SOBC2_2639 SOBC2_2597
     SOBC2_2593 SOBC2_2603 SOBC2_2566 SOBC2_2637 SOBC2_2586 SOBC2_2592
     SOBC2_2629 SOBC2_2569 SOBC2_2567 SOBC2_2625 SOBC2_2607 SOBC2_2613
     SOBC2_2574 SOBC2_2605 SOBC2_2663 SOBC2_2647 SOBC2_2564 SOBC2_2599
     SOBC2_2575 SOBC2_2661 SOBC2_2675 SOBC2_2560 SOBC2_2638 SOBC2_2636
     SOBC2_2654 SOBC2_2579 SOBC2_2619 SOBC2_2659 SOBC2_2610 SOBC2_2623
     SOBC2_2606 SOBC2_2601 SOBC2_2626 SOBC2_2618 SOBC2_2561 SOBC2_2644
     SOBC2_2635 SOBC2_2550 SOBC2_2602 SOBC2_2554 SOBC2_2616 SOBC2_2648
     SOBC2_2594 SOBC2_2630 SOBC2_2622 SOBC2_2736 SOBC2_2609 SOBC2_2627
     SOBC2_2728 SOBC2_2632 SOBC2_2587 SOBC2_2596 SOBC2_2723 SOBC2_2714
     SOBC2_2642 SOBC2_2176 SOBC2_2186 SOBC2_2108 SOBC2_2165 SOBC2_2174
     SOBC2_2205 SOBC2_2097 SOBC2_2094 ;

proc sort;
     by PID;



* Merging Gene expression matrix with Regressor matrix. ;

data horiz;
     merge regressors genes_horiz;
     by PID;

     * Removing gene expression cases with no covariate data available. ;

     if Study = . then delete;

proc sort;
     by PID Study Male Age White BMI Alcohol Smoke Ill CD3D CD3E CD4 CD8A
     CD19 FCGR3A NCAM1 CD14 Hedonic Eudaimonic PWB SWB Flourish TotalMHC
     BrownPWB BrownSWB;



* Transposing to stacked-Gene format;

proc transpose;
     var il1a il1b il6 il8 tnf ptgs1 ptgs2 fos fosb fosl1 fosl2 jun junb
     jund nfkb1 nfkb2 rel rela relb igj igll1 igll3 gbp1 ifi16 ifi27
     ifi27l1 ifi27l2 ifi30 ifi35 ifi44 ifi44l ifi6 ifih1 ifit1 ifit1l ifit2
     ifit3 ifit5 ifitm1 ifitm2 ifitm3 ifitm4p ifitm5 ifnb1 irf8 irf2 irf7
     mx1 mx2 oas1 oas2 oas3 oasl;
     by PID Study Male Age White BMI Alcohol Smoke Ill CD3D CD3E CD4 CD8A
     CD19 FCGR3A NCAM1 CD14 Hedonic Eudaimonic PWB SWB Flourish TotalMHC
     BrownPWB BrownSWB;

data vertical;
     set;

     Gene = _NAME_;

     * Log2-transform gene expression values. ;

     Expression = log2(COL1);

proc sort data=vertical;
     by Gene Study;


* Reverse sign of standardized expression values for negative elements of CTRA: Type I Interferon and Antibody genes. ;

data pooled;
     set vertical;

     if Gene ne 'IL1A'
     and Gene ne 'IL1B'
     and Gene ne 'IL6'
     and Gene ne 'IL8'
     and Gene ne 'TNF'
```

```
     and Gene ne 'PTGS1'
     and Gene ne 'PTGS2'
     and Gene ne 'FOS'
     and Gene ne 'FOSB'
     and Gene ne 'FOSL1'
     and Gene ne 'FOSL2'
     and Gene ne 'JUN'
     and Gene ne 'JUNB'
     and Gene ne 'JUND'
     and Gene ne 'NFKB1'
     and Gene ne 'NFKB2'
     and Gene ne 'REL'
     and Gene ne 'RELA'
     and Gene ne 'RELB'
     then Expression = -1*Expression;

proc sort;
     by PID Gene;


* Analyze data for Discovery Study only. ;

data study1;
     set pooled;

     * Removing data from Confirmation Study. ;

     if Study = 2 then delete;



* Standardizing continuous well-being scores for Discovery Study alone. ;

proc standard m=0 s=1 data=study1 out=study1_z;
     var Hedonic Eudaimonic;



data study1_regressors;
     set study1_z;

     * Remove redundant Gene-specific regressor submatrices within stacked data matrix. ;

     if Gene ne 'IL1B' then delete;



* Characterizing Discovery Study-specific regressor matrix. ;

*proc means data=study1_regressors n nmiss mean stddev min max;
*     title 'Discovery Study: Descriptive statistics for regressors';
*     var Male Age White BMI Alcohol Smoke Ill CD3D CD3E CD4 CD8A CD19
*     FCGR3A NCAM1 CD14 Hedonic Eudaimonic;


* Linear model analyses. ;

proc sort data=study1_z;
     by Gene;

ods select none;

proc reg data=study1_z;
     title 'Discovery Study: Eudaimonic & Hedonic well-being';
     title2 'Corrected GSE45330_Regressors';
     model Expression = Male Age White BMI Ill Alcohol Smoke
     CD3D CD3E CD4 CD8A CD19 FCGR3A NCAM1 CD14 Hedonic Eudaimonic;
     ods output ParameterEstimates=Coefficients1;
     by Gene;

data AssociationCoefficients1;
     set Coefficients1;
     if Variable ne 'Eudaimonic' and Variable ne 'Hedonic' then delete;

run;

ods select all;

proc sort data=AssociationCoefficients1;
     by Variable Gene;
```

```
proc means noprint data=AssociationCoefficients1;
     var Estimate;
     by Variable;
     output out=MeanCoefficients1 mean=MeanCoeff1;

data PctDif1;
     set MeanCoefficients1;
     PctDifPer4SD = 100*(-1+2**(4*MeanCoeff1));

proc print;
     var Variable PctDifPer4SD;



* Linear model analyses with errant white=4 value for case SOBC1_1299. ;

data study1_z2;
     set study1_z;

     if PID = 'SOBC1_1299' then White = 4; * regenerating coding error from original GSE45330_Regressors file;

ods select none;

proc reg data=study1_z2;
     title 'Discovery Study: Eudaimonic & Hedonic well-being';
     title2 'Original GSE45330_Regressors (with coding error SOBC1_1299 White=4)';
     model Expression = Male Age White BMI Ill Alcohol Smoke
     CD3D CD3E CD4 CD8A CD19 FCGR3A NCAM1 CD14 Hedonic Eudaimonic;
     ods output ParameterEstimates=Coefficients1b;
     by Gene;

data AssociationCoefficients1b;
     set Coefficients1b;
     if Variable ne 'Eudaimonic' and Variable ne 'Hedonic' then delete;

run;

ods select all;

proc sort data=AssociationCoefficients1b;
     by Variable Gene;

proc means noprint data=AssociationCoefficients1b;
     var Estimate;
     by Variable;
     output out=MeanCoefficients1b mean=MeanCoeff1b;

data PctDif1b;
     set MeanCoefficients1b;
     PctDifPer4SD = 100*(-1+2**(4*MeanCoeff1b));

proc print;
     var Variable PctDifPer4SD;



data study2;
     set pooled;

     * Removing data from Discovery Study. ;

     if Study = 1 then delete;

     * Removing missing IL6 data = all IL6 data in Confirmation Study. ;

     if Gene = 'IL6' and Expression = . then delete;



* Standardizing continuous well-being scores for Confirmation Study alone. ;

proc standard m=0 s=1 data=study2 out=study2_z;
     var Hedonic Eudaimonic;



data study2_regressors;
     set study2_z;
```

```
     * Remove redundant Gene-specific regressor submatrices within stacked data matrix. ;

     if Gene ne 'IL1B' then delete;



* Characterizing Confirmation Study-specific regressor matrix. ;

*proc means data=study2_regressors n nmiss mean stddev min max;
*     title 'Confirmation Study: Descriptive statistics for regressors';
*     var Male Age White BMI Alcohol Smoke Ill CD3D CD3E CD4 CD8A CD19
*     FCGR3A NCAM1 CD14 Hedonic Eudaimonic;


* Linear model analyses. ;

proc sort data=study2_z;
     by Gene;

ods select none;

proc reg data=study2_z;
     title 'Confirmation Study: Eudaimonic & Hedonic well-being';
     title2 ' ';
     model Expression = Male Age White BMI Ill Alcohol Smoke
     CD3D CD3E CD4 CD8A CD19 FCGR3A NCAM1 CD14 Hedonic Eudaimonic;
     ods output ParameterEstimates=Coefficients2;
     by Gene;

data AssociationCoefficients2;
     set Coefficients2;
     if Variable ne 'Eudaimonic' and Variable ne 'Hedonic' then delete;

run;

ods select all;

*proc print data=AssociationCoefficients2;

proc sort data=AssociationCoefficients2;
     by Variable Gene;

proc means noprint data=AssociationCoefficients2;
     var Estimate;
     by Variable;
     output out=MeanCoefficients2 mean=MeanCoeff2;

data PctDif2;
     set MeanCoefficients2;
     PctDifPer4SD = 100*(-1+2**(4*MeanCoeff2));

proc print;
     var Variable PctDifPer4SD;
```
